# Metabolic-GWAS provides insights into genetic architecture of seed metabolome in buckwheat

**DOI:** 10.1186/s12870-023-04381-x

**Published:** 2023-07-28

**Authors:** Sajad Majeed Zargar, Madhiya Manzoor, Basharat Bhat, Amir Bashir Wani, Parvaze Ahmad Sofi, Jebi Sudan, Leonard Barnabas Ebinezer, Stefano Dall’Acqua, Gregorio Peron, Antonio Masi

**Affiliations:** 1 Proteomics Laboratory, Division of Plant Biotechnology, Sher-E-Kashmir University of Agricultural Sciences and Technology of Kashmir, Shalimar, Srinagar, Jammu and Kashmir, India; 2 Division of Animal Biotechnology, Sher-E-Kashmir University of Agricultural Sciences and Technology of Kashmir, Srinagar, India; 3 Division of Genetics and Plant Breeding, Sher-E-Kashmir University of Agricultural Sciences and Technology of Kashmir, Srinagar, India; 4 Department of Agronomy, Food, Natural Resources, Animals, and Environment, University of Padova, Padua, Italy; 5 Department of Pharmaceutical and Pharmacological Sciences, University of Padova, Padua, Italy; 6 Department of Molecular and Translational Medicine (DMMT), University of Brescia, Brescia, Italy

**Keywords:** Buckwheat, Metabolomics, GBS, QTLs, Candidate genes, Metabolites

## Abstract

**Background:**

Buckwheat (*Fagopyrum* spp.), belonging to the Polygonaceae family, is an ancient pseudo-cereal with high nutritional and nutraceutical properties. Buckwheat proteins are gluten-free and show balanced amino acid and micronutrient profiles, with higher content of health-promoting bioactive flavonoids that make it a golden crop of the future. Plant metabolome is increasingly gaining importance as a crucial component to understand the connection between plant physiology and environment and as a potential link between the genome and phenome. However, the genetic architecture governing the metabolome and thus, the phenome is not well understood. Here, we aim to obtain a deeper insight into the genetic architecture of seed metabolome in buckwheat by integrating high throughput metabolomics and genotyping-by-sequencing applying an array of bioinformatics tools for data analysis.

**Results:**

High throughput metabolomic analysis identified 24 metabolites in seed endosperm of 130 diverse buckwheat genotypes. The genotyping-by-sequencing (GBS) of these genotypes revealed 3,728,028 SNPs. The Genome Association and Prediction Integrated Tool (GAPIT) assisted in the identification of 27 SNPs/QTLs linked to 18 metabolites. Candidate genes were identified near 100 Kb of QTLs, providing insights into several metabolic and biosynthetic pathways.

**Conclusions:**

We established the metabolome inventory of 130 germplasm lines of buckwheat, identified QTLs through marker trait association and positions of potential candidate genes. This will pave the way for future dissection of complex economic traits in buckwheat.

**Supplementary Information:**

The online version contains supplementary material available at 10.1186/s12870-023-04381-x.

## Background

Buckwheat (*Fagopyrum* spp.) is a pseudo-cereal belonging to the Polygonaceae family. The genus *Fagopyrum* contains 27 species, of which two diploid species—Tartary buckwheat (*Fagopyrum tataricum*) and common buckwheat (*Fagopyrum esculentum*) are grown for food [1]. These two species vary in their breeding system, *F. tataricum* being self-fertile mainly perform inbreeding, while *F. esculentum* is an insect-pollinated, and obligate out breeder [2]. The grain is consumed after boiling or steaming, or powdered into gluten-free flour.

Buckwheat is a valuable health-promoting crop that is used for preparing number of functional foods and nutraceuticals products. It has high-quality, gluten-free proteins as well as a wealth of bioactive ingredients and antioxidants. These characteristics account for its current high demand [3]. The balanced essential amino acids, resistant starch, vitamins, and minerals available in buckwheat are beneficial to human health. Additionally, it is a rich source of bioactive flavonoids like epicatechin, rutin, quercetin, and (iso)vitexin, all of which have been shown to have favourable effects on hyperlipidaemia, diabetes, and vascular diseases [4–6]. In particular, Tartary buckwheat is abundant in rutin, a citrus flavonoid that helps the body to use vitamin C, produce collagen and strengthen blood vessels [7].

In view of the increasing interest in nutraceutical crops as potential candidates for crop diversification and a shift from food to nutritional and health security, a large scale nutri-genomic investigations across crops has been initiated, in order to unravel the potential of underutilised species as frontline foods to promote health [8]. Despite its obvious potential as a functional food, buckwheat has not been fully harnessed due to low yield, self-incompatibility, increased seed cracking, limited seed set, lodging, and frost susceptibility. To overcome these bottlenecks, there is a need to improve these traits in this underutilized species [9]. The availability of diverse data, as well as the population structure of crop germplasm, will be valuable genetic resources for discovering genes in buckwheat for improving it as a potential crop for the future [10].

In-depth research on the flavonoid biosynthesis pathway in model plants and several crop species has recently gained attention. These available experimental evidences indicate that metabolomics can provide novel insights into the biosynthesis pathways, especially in crops that possesses high value traits associated with bioactive antioxidant metabolites. Buckwheat being well known for such traits is attracting high throughput omics research in the recent years. Metabolome Based Genome Wide Association Study (mGWAS) or metabolomic quantitative trait loci (mQTL) mapping, in particular, is emerging as a powerful tool for mining the genetic loci contributing to metabolite diversification. Furthermore, the relationship of these metabolites, naturally deputed to defence against biotic and abiotic stress [11] with food quality and flavour has been thoroughly investigated [12]. However, to the best of our knowledge, no mGWAS or mQTL studies in buckwheat have been reported till date. With the advancement of genome sequencing and bioinformatics technologies, approaches such as association and linkage mapping have come a long way to unravel the genetic diversity of targeted traits across crops. Furthermore, plant association mapping has revealed new genetic and biochemical information about metabolomes. Genome-wide association studies (GWAS) are frequently used to identify new genes and QTLs by locating significant allelic differences in candidate genes underpinning quantitative and complicated traits, such as those linked to growth, development, stress tolerance, and nutritional quality [13]. As a potential technique for moving forward, mGWAS entails merging genotyping and metabolome data from the diverse crop germplasm [14], with a striking example from highland barley (Qingke), mGWAS study has been carried out for mining genes involved in phenylpropane metabolic pathway [15].

Considering no such information is available till date on buckwheat, in the present study, high throughput metabolomics analysis was carried out on the seed endosperm of 130 diverse buckwheat genotypes and GBS-based SNP genotyping was performed to identify marker trait associations. Further, candidate genes located in 100 kb region of known QTLs were identified.

## Results

### Identification of phenolic metabolites in buckwheat extracts

Results obtained from HPLC–DAD-MS characterisation are reported in Supplementary Table 1, and a representative chromatogram is also shown (Supplementary Fig. 1). Among the identified metabolites, 10 were flavonoids (both glycosidic derivatives and aglycones), 6 were phenolic acids (benzoic, caffeic, and ferulic acids derivatives), 3 were catechin derivatives, and 3 were gallic acid derivatives. Among the flavonoids, Rutin and its aglycone (quercetin) possess numerous biological activities. Exposure of buckwheat grain to moisture results in enzymatic breakdown of rutin to quercetin by rutinosidase, as it also happens after milling and mixing of the flour with water. The beneficial effect of rutin depends upon its concentration in the final product that further depends upon the conversion rate of rutin to quercetin. Thus, the quantification of both rutin and quercetin are considered at seed level. Out of 22 different metabolites, 18 were found to be significantly associated with different SNP markers (Table 1).

**Table 1:** Details of significant QTLs associated with seed metabolome content

Trait	Chr. no	Position	*p*-value	R^2^	Effect	Gene	Genic Location	Nucleotide Change using *Fagopyrum tataricum* as reference genome	Nucleotide change using *Fagopyrum esculentum* as reference genome
**DH**	6	31,943,608	1.85E-05	0.158705	-0.02714	FtPinG0003273700.01-FtPinG0009352600.01	intergenic_region	G > C	C
	6	31,943,643	5.08E-05	0.141664	-0.02666	FtPinG0003273700.01-FtPinG0009352600.01	intergenic_region	**C > T**	**C > T**
	7	1,442,391	1.73E-04	0.121484	-0.01428	FtPinG0002741000.01-FtPinG0002741100.01	intergenic_region	**T > G**	**T > G**
	7	1,442,405	1.12E-04	0.128641	-0.0152	FtPinG0002741000.01-FtPinG0002741100.01	intergenic_region	**T > G**	**C > G**
	7	1,442,440	7.07E-05	0.136179	-0.01541	FtPinG0002741000.01-FtPinG0002741100.01	intergenic_region	**A > C**	**T > C**
**CAH**	1	686,973	1.43E-04	0.140211	0.013776	FtPinG0000109300.01	transcript	**A > G**	**T > G**
	2	20,704,365	1.91E-04	0.135583	0.009183	FtPinG0007484300.01	transcript	**T > G**	**C > G**
	3	27,868,660	9.36E-05	0.147047	0.013873	FtPinG0004748800.01-FtPinG0004818900.01	intergenic_region	**C > T**	**C > T**
**FARD**	1	690,702	1.11E-04	0.247833	0.049632	FtPinG0000107500.01	transcript	C > T	T
	1	690,741	1.47E-04	0.243917	0.045342	FtPinG0000107500.01	transcript	C > T	T
	1	24,428,872	1.32E-04	0.245393	-0.02166	FtPinG0009765900.01-FtPinG0009765800.01	intergenic_region	A > G	G
	1	36,893,765	1.30E-04	0.245693	-0.02427	FtPinG0009723600.01-FtPinG0009723000.01	intergenic_region	**G > A**	**G > A**
	1	56,206,146	7.86E-06	0.286407	-0.03312	FtPinG0009546600.01-FtPinG0009547000.01	intergenic_region	**T > C**	**A > C**
	1	56,206,255	7.66E-06	0.286781	-0.02978	FtPinG0009546600.01-FtPinG0009547000.01	intergenic_region	C > G	G
	1	56,206,256	1.46E-05	0.277167	-0.02798	FtPinG0009546600.01-FtPinG0009547000.01	intergenic_region	**T > G**	**A > G**
	1	66,342,001	1.61E-05	0.275742	0.05765	FtPinG0009047000.01	transcript	G > A	A
	1	66,350,397	3.16E-05	0.265903	0.052706	FtPinG0009047800.01	transcript	**A > C**	**T > C**
	1	66,772,599	5.13E-05	0.258913	0.050667	FtPinG0008819900.01	transcript	T > C	C
	1	66,772,612	8.22E-05	0.252153	0.047167	FtPinG0008819900.01	transcript	**C > A**	**G > A**
	1	67,240,863	6.82E-05	0.254816	-0.02415	FtPinG0007913300.01-FtPinG0007914400.01	intergenic_region	C > A	A
	2	17,630,572	6.70E-05	0.255069	-0.02232	FtPinG0006267400.01	transcript	**C > T**	**C > T**
	2	43,827,225	9.62E-05	0.249909	-0.02301	FtPinG0007781400.01-FtPinG0007781500.01	intergenic_region	**A > T**	**C > T**
	3	24,391,155	1.26E-04	0.246074	-0.02141	FtPinG0008451400.01-FtPinG0007098800.01	intergenic_region	C > T	T
	3	24,391,204	1.26E-04	0.246059	-0.02214	FtPinG0008451400.01-FtPinG0007098800.01	intergenic_region	C > T	T
	3	24,391,246	3.72E-05	0.263542	-0.02454	FtPinG0008451400.01-FtPinG0007098800.01	intergenic_region	C > T	T
	3	27,527,353	8.21E-05	0.25216	-0.02363	FtPinG0008424800.01-FtPinG0006577900.01	intergenic_region	T > C	C
	4	15,679,429	1.68E-04	0.242033	-0.02163	FtPinG0004200100.01-FtPinG0005928300.01	intergenic_region	**G > A**	**C > A**
	4	40,483,173	8.51E-05	0.251657	-0.02554	FtPinG0008592100.01-FtPinG0007101500.01	intergenic_region	**G > A**	**T > A**
	4	40,483,242	1.01E-04	0.24915	-0.02325	FtPinG0008592100.01-FtPinG0007101500.01	intergenic_region	**C > T**	**A > T**
	6	21,277,090	1.99E-04	0.239631	-0.02061	FtPinG0009334000.01	transcript	C > A	A
	6	25,196,690	1.27E-04	0.245935	-0.02207	FtPinG0006368000.01-FtPinG0006368600.01	intergenic_region	G > A	A
	6	33,349,635	8.44E-07	0.320425	-0.03594	FtPinG0009101100.01-FtPinG0006198100.01	intergenic_region	**C > A**	**T > A**
	8	27,024,873	1.47E-04	0.243868	-0.02266	FtPinG0009096400.01-FtPinG0009096700.01	intergenic_region	**G > A**	**T > A**
	8	27,168,605	1.28E-04	0.245854	-0.02628	FtPinG0009099100.01-FtPinG0007437200.01	intergenic_region	**C > T**	**C > T**
	8	31,903,089	6.55E-05	0.255389	-0.02226	FtPinG0007066900.01-FtPinG0007066500.01	intergenic_region	**T > C**	**T > C**
**CG**	3	5,955,778	1.56E-04	0.147546	0.018385	FtPinG0007345300.01-FtPinG0007345100.01	intergenic_region	**G > A**	**G > A**
	6	40,712,816	1.84E-04	0.144954	-0.01958	FtPinG0000951700.01-FtPinG0009389800.01	intergenic_region	**C > T**	**A > T**
	6	47,601,958	1.67E-04	0.146488	-0.02541	FtPinG0009744500.01-FtPinG0009745000.01	intergenic_region	**G > A**	**C > A**
**SI**	3	27,531,299	1.64E-04	0.138372	-0.00649	FtPinG0008424800.01-FtPinG0006577900.01	intergenic_region	**C > G**	**A > G**
	4	18,035,789	1.86E-04	0.136442	-0.00486	FtPinG0005139500.01-FtPinG0003394400.01	intergenic_region	**C > T**	**C > T**
	5	9,638,542	6.62E-05	0.153038	-0.00541	FtPinG0009308500.01-FtPinG0009309300.01	intergenic_region	**A > G**	**T > G**
	5	19,139,130	2.00E-04	0.135279	-0.00455	FtPinG0006720000.01	transcript	C > T	T
	5	52,022,751	1.64E-04	0.138378	-0.00575	FtPinG0000759400.01-FtPinG0001108200.01	intergenic_region	**G > A**	**G > A**
**Catechin**	1	37,667,295	1.79E-04	0.186122	0.007466	FtPinG0003113900.01-FtPinG0008771600.01	intergenic_region	**C > A**	**C > A**
	3	5,955,359	8.79E-06	0.232852	0.009372	FtPinG0007345300.01	transcript	C > T	T
	3	5,955,370	4.95E-05	0.205691	0.00858	FtPinG0007345300.01	transcript	**A > T**	**A > T**
	3	5,955,386	3.91E-05	0.209333	0.008408	FtPinG0007345300.01	transcript	**T > A**	**T > A**
	4	47,183,385	1.11E-04	0.193272	-0.00794	FtPinG0005258100.01-FtPinG0007507100.01	intergenic_region	G > A	A
	5	20,516,429	1.37E-04	0.190159	-0.00876	FtPinG0006758300.01-FtPinG0007298300.01	intergenic_region	**C > A**	**T > A**
	6	3,469,009	1.97E-04	0.184654	-0.00775	FtPinG0002700100.01	transcript	**G > A**	**T > A**
**UNK2**	2	30,282,729	6.59E-05	0.171165	-0.00877	FtPinG0006957600.01-FtPinG0009282100.01	intergenic_region	**T > A**	**C > A**
**EAEC**	5	9,852,061	4.64E-05	0.264065	-0.01507	FtPinG0009313800.01	transcript	C > T	T
**Vanillin**	1	770,266	1.81E-04	0.20833	-0.00373	FtPinG0000116900.01	transcript	**T > C**	**T > C**
	2	25,219,312	1.36E-04	0.212469	-0.00412	FtPinG0008771000.01-FtPinG0008770600.01	intergenic_region	G > A	A
	4	32,201,379	1.50E-04	0.211102	-0.00393	FtPinG0002498500.01-FtPinG0007295400.01	intergenic_region	**C > T**	**C > T**
	4	37,569,986	3.56E-05	0.232479	-0.00379	FtPinG0005694200.01-FtPinG0005693900.01	intergenic_region	G > A	A
	5	48,972,415	1.62E-04	0.209901	-0.00353	FtPinG0006470900.01	transcript	T > G	G
	7	16,971,070	6.12E-05	0.22436	-0.00379	FtPinG0007389500.01	transcript	**C > T**	**G > T**
	7	26,624,210	4.57E-05	0.228734	-0.00496	FtPinG0008373300.01-FtPinG0006832800.01	intergenic_region	**G > A**	**C > A**
**Orientin**	5	20,236,700	1.59E-04	0.177486	-0.03427	FtPinG0006758900.01-FtPinG0006758300.01	intergenic_region	**C > T**	**N > T**
	8	44,828,457	3.37E-05	0.20154	-0.04085	FtPinG0004158200.01	transcript	**G > C**	**G > C**
	8	44,828,469	1.18E-04	0.182103	-0.03586	FtPinG0004158200.01	transcript	**T > G**	**A > G**
**Rutin**	3	18,109,943	1.88E-04	0.12706	-0.0253	FtPinG0001983600.01	transcript	**C > G**	**A > G**
	5	52,022,270	3.11E-05	0.156608	-0.03027	FtPinG0000759400.01-FtPinG0001108200.01	intergenic_region	**G > A**	**G > A**
**Duratin**	1	41,194,007	3.84E-05	0.252683	0.020089	FtPinG0009552900.01-FtPinG0009553100.01	intergenic_region	**T > C**	**G > C**
	1	61,107,764	1.18E-04	0.236442	-0.01614	FtPinG0004992900.01	transcript	**G > T**	**A > T**
	3	25,220,994	1.20E-04	0.23612	-0.02378	FtPinG0000957700.01-FtPinG0000705900.01	intergenic_region	**C > A**	**T > A**
	5	20,432,271	7.80E-05	0.242379	-0.02215	FtPinG0006758300.01-FtPinG0007298300.01	intergenic_region	**G > A**	**T > A**
**QDG**	1	36,305,222	1.88E-04	0.121977	-0.01995	FtPinG0008486000.01-FtPinG0002428800.01	intergenic_region	C > A	A
	6	33,447,847	1.11E-04	0.130612	-0.03464	FtPinG0009101100.01-FtPinG0006198100.01	intergenic_region	**G > A**	**C > A**
	7	36,838,229	1.87E-05	0.160296	-0.01753	FtPinG0001130600.01-FtPinG0006711700.01	intergenic_region	**A > G**	**C > G**
	7	36,838,233	8.32E-06	0.174117	-0.01802	FtPinG0001130600.01-FtPinG0006711700.01	intergenic_region	**A > G**	**C > G**
**EEM**	1	56,206,007	1.70E-04	0.230017	0.018241	FtPinG0009546600.01-FtPinG0009547000.01	intergenic_region	C > A	A
**EED**	1	36,902,263	8.49E-05	0.140581	-0.15143	FtPinG0009723000.01	transcript	**G > A**	**G > A**
	1	40,494,217	6.52E-05	0.144906	-0.15095	FtPinG0009459500.01-FtPinG0006043600.01	intergenic_region	C > T	T
	1	56,205,855	9.67E-05	0.138464	-0.14649	FtPinG0009546600.01-FtPinG0009547000.01	intergenic_region	**C > T**	**G > T**
	1	56,205,857	4.99E-05	0.149315	-0.15454	FtPinG0009546600.01-FtPinG0009547000.01	intergenic_region	A > G	G
	1	63,055,659	4.62E-05	0.150599	-0.15261	FtPinG0007147500.01-FtPinG0007147100.01	intergenic_region	T > C	A
	2	24,116,413	1.53E-04	0.131037	-0.14383	FtPinG0009268700.01	transcript	**G > A**	**C > A**
	4	22,592,618	1.99E-04	0.126772	-0.12859	FtPinG0006817900.01-FtPinG0006817800.01	intergenic_region	**C > T**	**A > T**
	4	27,546,189	1.12E-04	0.136066	-0.14038	FtPinG0006589600.01-FtPinG0001650100.01	intergenic_region	C > T	T
	4	37,570,298	1.62E-04	0.130047	-0.14167	FtPinG0005694200.01-FtPinG0005693900.01	intergenic_region	T > C	C
	5	50,760,514	1.50E-04	0.131302	-0.14217	FtPinG0009566500.01-FtPinG0009566800.01	intergenic_region	**A > G**	**C > G**
	7	40,000,586	1.25E-04	0.134232	-0.14186	FtPinG0005061300.01-FtPinG0005842300.01	intergenic_region	**T > A**	**T > A**
**GEDC**	1	19,115,204	1.02E-04	0.158153	0.003964	FtPinG0006187600.01-FtPinG0006187400.01	intergenic_region	**C > T**	**G > T**
	3	21,354,743	1.11E-04	0.156796	0.005107	FtPinG0007606600.01-FtPinG0007633300.01	intergenic_region	**T > A**	**C > A**
	7	5,447,892	1.94E-04	0.148009	0.003523	FtPinG0008977900.01-FtPinG0008978100.01	intergenic_region	A > T	T
**Quercetin**	2	6,522,591	5.37E-05	0.189762	-0.61128	FtPinG0006973300.01-FtPinG0006973100.01	intergenic_region	**T > A**	**T > A**
	2	19,936,435	8.33E-05	0.182878	-0.60977	FtPinG0000053600.01-FtPinG0005240800.01	intergenic_region	**G > A**	**G > A**
	2	19,942,252	1.88E-04	0.170308	-0.6232	FtPinG0000053600.01-FtPinG0005240800.01	intergenic_region	**C > T**	**C > T**
	2	35,787,786	6.26E-05	0.187339	-0.66247	FtPinG0004834600.01-FtPinG0004835000.01	intergenic_region	**A > G**	**T > T**
	2	47,754,251	1.91E-04	0.170036	-0.44249	FtPinG0002929100.01	transcript	G > T	T
	4	46,063,806	1.83E-04	0.170666	-0.56031	FtPinG0008652100.01	transcript	**G > A**	**N > A**
	5	29,043,577	1.01E-05	0.21647	-0.92787	FtPinG0006381600.01	transcript	**T > C**	**A > C**
	5	29,043,597	1.28E-04	0.17622	-0.73951	FtPinG0006381600.01	transcript	**T > C**	**G > C**
	7	23,524,375	1.64E-04	0.172378	-0.47712	FtPinG0001268600.01-FtPinG0001269100.01	intergenic_region	**G > A**	**C > A**
	8	24,957,491	1.85E-04	0.17055	-0.647	FtPinG0009120700.01-FtPinG0007897200.01	intergenic_region	**G > A**	**T > A**
**Kaempherol**	1	36,305,386	7.01E-05	0.176993	0.044452	FtPinG0008486000.01-FtPinG0002428800.01	intergenic_region	A > T	T
	5	29,043,577	1.80E-05	0.198776	-0.06374	FtPinG0006381600.01	transcript	**T > C**	**A > C**

### Metabolite fingerprinting of buckwheat samples

**Fig. 1:**
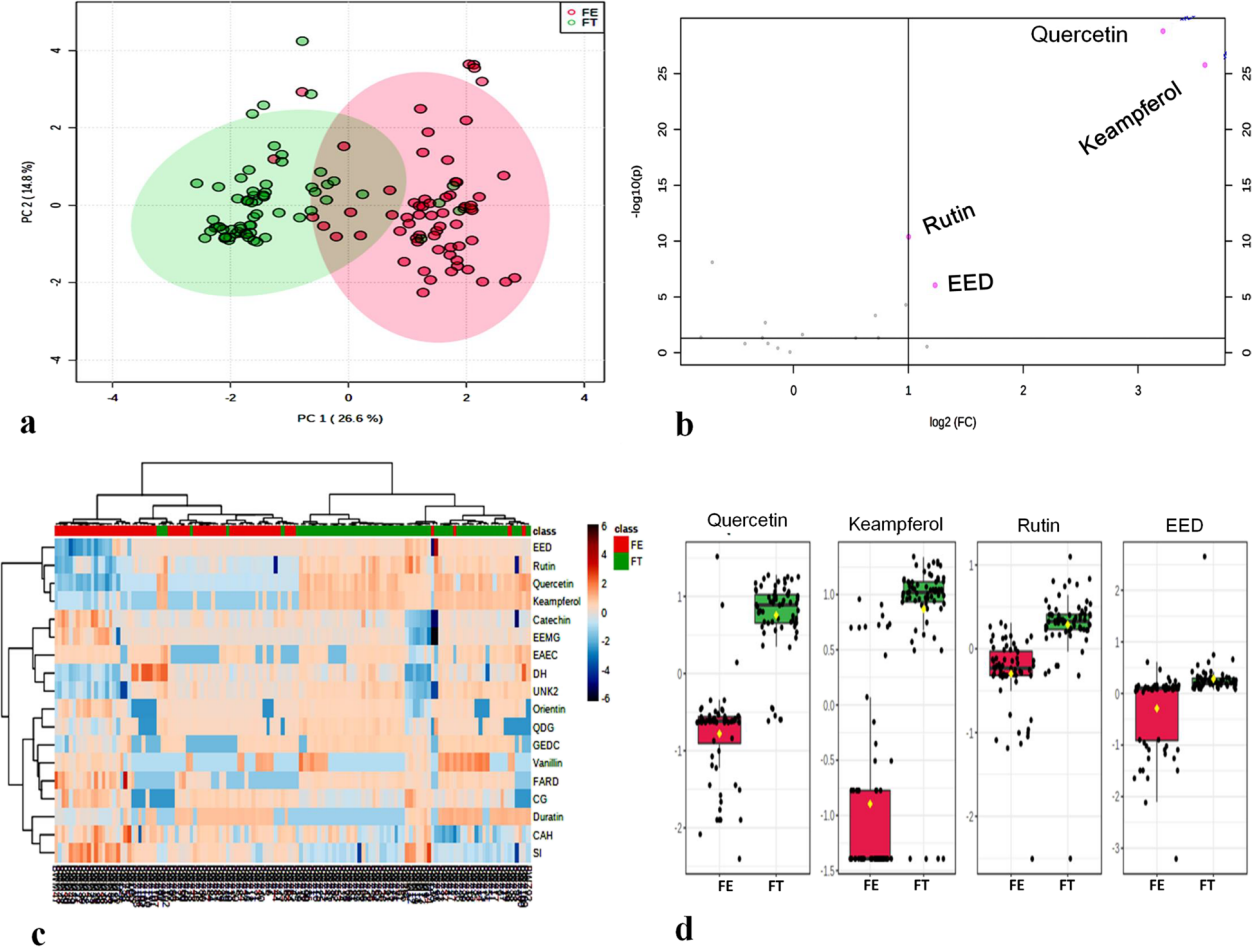
Metabolomic analysis of buckwheat samples**.** **a** PCA score plot. Red dots: *F. esculentum*; green dots: *F. tataricum*; (**b**) Volcano plot showing variables significantly associated to the two buckwheat sample groups. Variables on the right side of the plot are more abundant in *F. tataricum*, while those on the left side are more abundant in *F. esculentum*. Only variables significantly (FDR-*p* < 0.05) associated to one of the two groups are highlighted; (**c**) Heatmap plot. Red: *F. esculentum*; green: *F. tataricum*; (**d**) Boxplots showing the comparison of the amounts of significant variables in the two groups of buckwheat samples. Red: *F. esculentum*; Green: *F. tataricum*

Raw HPLC–DAD-MS data were analysed using multivariate statistics. An explorative analysis was performed, namely PCA. Results, reported in Fig. 1a, showed a clear distinction between *F. tataricum* and *F. esculentum* samples, indicating different metabolome compositions. The same group’s distinction can be observed from the heatmap in Fig. 1c. PCA score plot revealed that the variance explained by PC1 is higher than that explained by PC2 (26.6% vs. 14.8%), indicating that the inter-group variability is higher than the intra-group one.

Variables significantly associated with the two sample groups were selected using a volcano plot, where variables with FDR-adjusted *p*-value < 0.05 and with Fold Change > 2 were considered as significant descriptors. As can be observed in Fig. 1b, four variables were significantly associated to the *F. tataricum* samples group, namely rutin, quercetin, kaempferol and epiafzelchin-epicatechin-O-dimethylgallate (EED). The amounts of these variables in the two groups of samples are shown in Fig. 1d.

### Characterization and distribution of SNPs in buckwheat

**Fig. 2:**
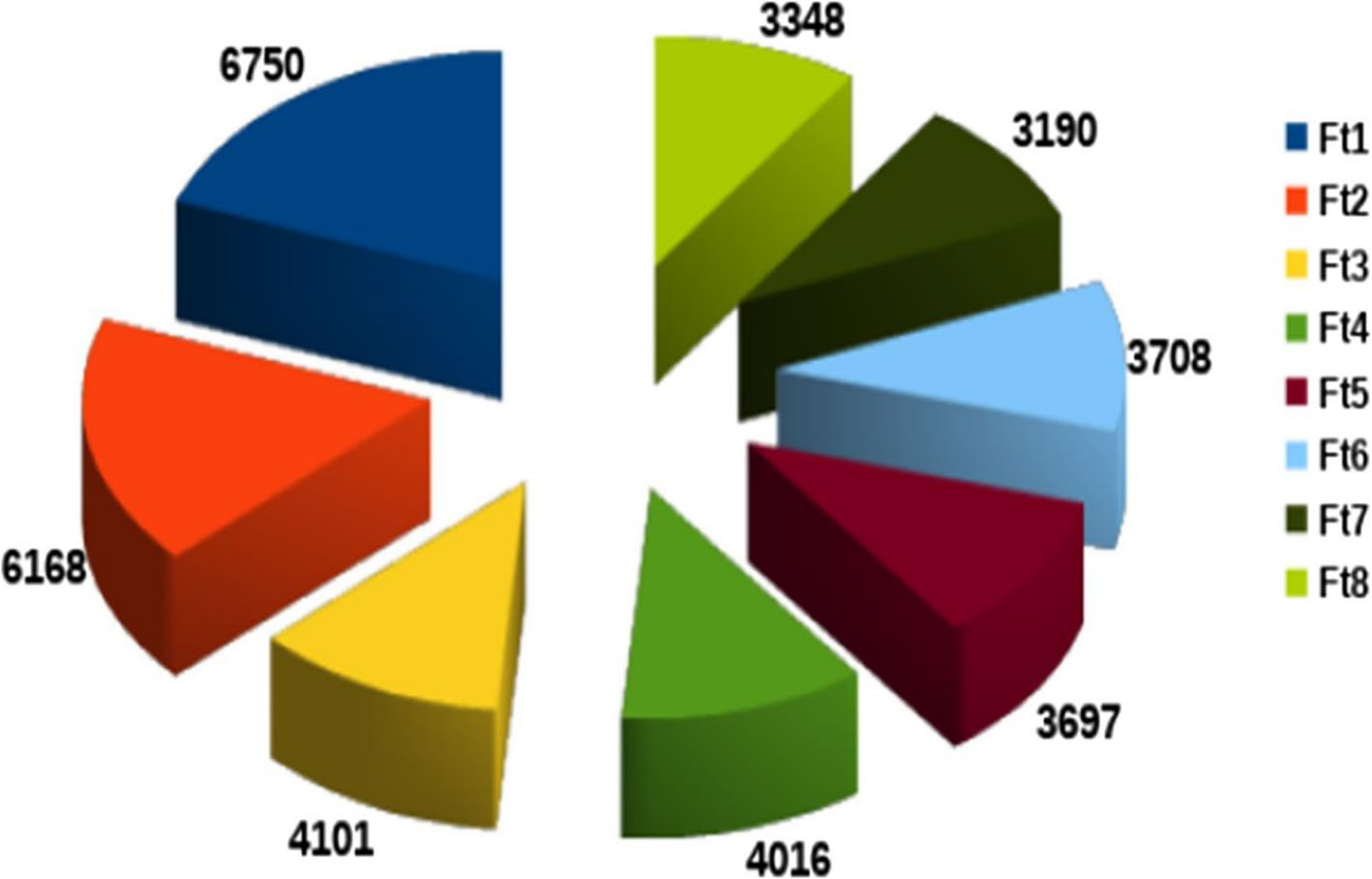
Chromosome wise SNP distribution in buckwheat germplasm

The data from the sequencing platform (with a sequencing depth of 121x) represent an average of 1.58 million reads per sample with a read length of 150 base pairs. The reads were then mapped to the buckwheat reference genome (GCA 002319775.1; http://www.mbkbase.org/Pinku1/) with an average mapping percentage of 90.78. The mapping of reads resulted in the identification of 4,142,684 variants, containing 3,728,028 SNPs and 414,656 InDels (214,798 insertions and 199,858 deletions). However, while considering 5% minor allele frequency and 30% missing rate, a total of 34,978 SNPs were observed. The chromosome wise distribution of SNP is shown in Fig. 2. The highest number of filtered SNPs (6750) was observed on chromosome (chr.) 1, whereas the lowest number of SNPs (3190) was found on chr. 7. SNPs have also been classified as having a high, low, moderate, or modifier impact, with the percentage of high being 0.413%, low being 1.566%, moderate being 2.105%, and modifier being 95.916%. According to the effects by functional class missense percent was 59.93%, nonsense was 3.37% and silent was 36.70%. Number of effects by type and region are mentioned in detail in Supplementary Table 2. Total number of transitions and transversions were found to be 39,416,882 and 23,590,338 respectively, with a transition by transversion ratio (Ts/Tv) of 1.6709.

In order to ensure similarity between two genomes of buckwheat, we performed pairwise alignment of both genomes using the GSALIGN program and visualized the result using DotPlot. Surprisingly from genome alignment, the major scaffolds from the common buckwheat genome aligned strongly with the Tataricum genome (Supplementary Fig. 2). So, we concluded that utilizing the Tataricum genome as a reference genome for annotating traits of common buckwheat doesn’t drift the overall study. It was further validated by using the common buckwheat genome (https://doi.org/10.1111/jipb.13459) [16] as reference genome to revalidate the already identified SNPs. We found a total of 68 QTLs (~ 67%) common among both genomes as detailed in Table 1. Moreover 31QTLs have the same residues in *Fagopyrum esculentum* genome as the genotypes under study.

### Genetic diversity and population structure

**Fig. 3:**
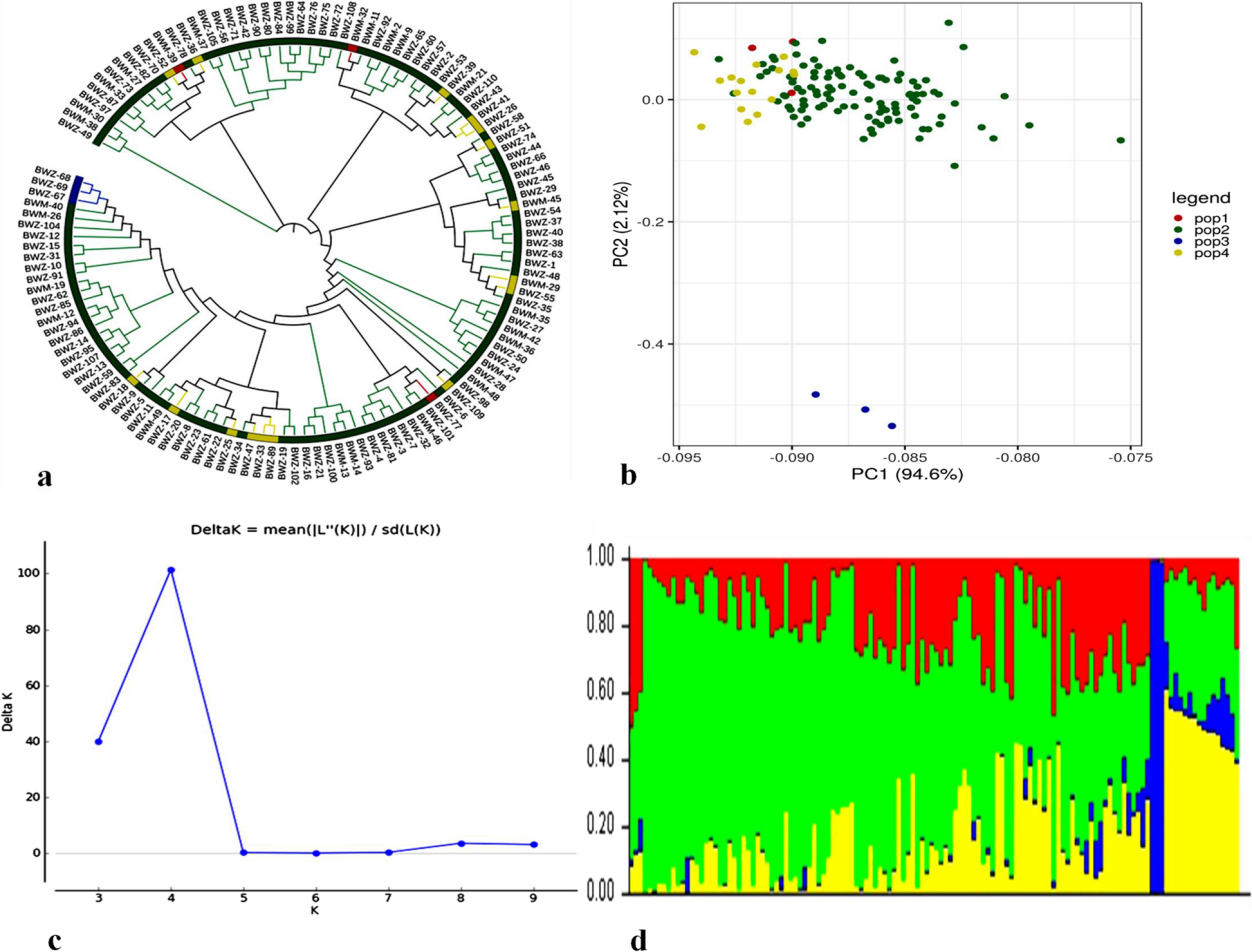
SNP markers based population analysis. **a** UPGMA dendrogram showing genetic relationship among 130 genotypes; (**b**) PCA Plot of Buckwheat genotypes; (**c**) Peak of delta K; (**d**) structure analysis indicated genotypes grouping into four sub-populations based on membership coefficients indicated on vertical coordinate

All paired genetic distances between the 130 buckwheat lines in this study were calculated using SNP-based genotypic data. A neighbouring tree revealed that the genotypes were divided into four main groups, which were further subdivided based on the genetic distances (Fig. 3a). Dendrogram analysis revealed that among the four major groups there was one minor group which was clustered together and included only three genotypes, i.e. BWM30, BWM38 and BWZ49. These three genotypes were all from the same species, *F. esculentum*, and are thus closely related. PCA revealed variations among buckwheat genotypes (Fig. 3b). Furthermore, the population structure was scored for K values ranging from 1 to 12 across the panel using high quality SNPs in population structure analysis. The delta K peak was found to be the highest at K = 4, and 130 buckwheat genotypes were classified into four populations (Fig. 3c and d). Furthermore, this was consistent with the neighbour-joining tree with only minor deviations.

### Marker trait association

**Fig. 4:**
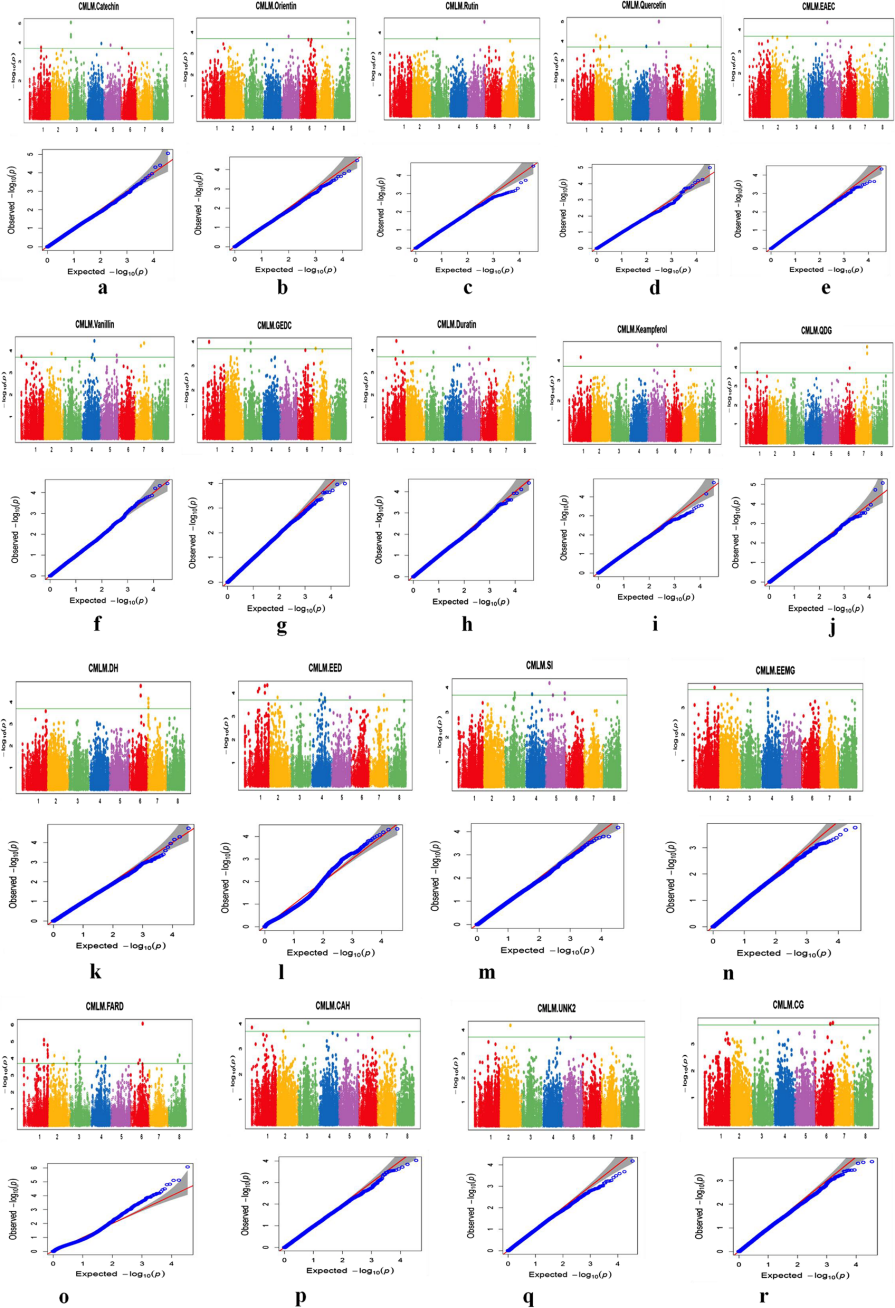
The figure showing the Manhattan plots and Q-Q plots of different metabolites. **a** Catechin, (**b**) Orientin, (**c**) Rutin, (**d**) Quercetin, (**e**) (epi)afzelchin-(epi)catechin, (**f**) Vanillin, (**g**) Galloyl ester of 5,6,7-trihydroxy-2,3 dihydrocyclopents (**b**)chromene-1,9-dione-3-carboxylic acid hexoside, (**h**) Duratin, (**i**) Keampferol, (**j**) Quercetin, 3-D-glucoside, (**k**) Diacaffeoyl-hexoside, (**l**) Epiafzelchin epicatechin-o-dimethylgallate, (**m**) Swertiamacroside isomer, (**n**) Epiafzelchin epicatechin-o-methylgallate, (**o**) Ferulic acid rhamnosyl derivative, (**p**) Caffeixc acid hexose, (**q**) UNK2 and (**r**) Catechin Glycoside

A total of 99 markers were found to be having significant association with 18 metabolites. The details of these marker trait associations are summarized in Table 1 and depicted in Manhattan and QQ-Plots (Fig. 4 A-R). GWAS was performed for 18 buckwheat seed metabolites (catechin, orientin, rutin, quercetin, EAEC, vanillin, GEDC, duratin, keampferol, QDG, DH, EED, SI, EEMG, FARD, CAH, UNK2 and CG). The analysis was done using GAPIT CMLM method. GAPIT uses FDR adjusted p-value to filter significant SNPs. This approach reduces chances of false positive SNP makers in further analysis. In Manhattan plot dotted green line shows *p*-value and solid green line shows FDR adjusted p-value. Out of total 3,728,028 SNPs, 34,978 were found to be significantly associated with different seed metabolites with 12.14–32.04% phenotypic variance. A total of 27 SNPs were found significantly associated with the metabolite ferulic acid rhamnosyl derivative (FARD). Out of 27, 12 SNPs were found in chr. 1; as such this region could be regarded as hot-spot of SNPs for this particular metabolite. One SNP on chr.6 (*p*-value** = **8.44E-07) contributed for 32.04% of phenotypic variation. For metabolite epiafzelchin epicatechin-o-dimethylgallate (EED), 11 SNPs were significantly associated. These were positioned on chr.1, chr.2, chr.4, chr.5, and chr.7 with the highest number of SNPs (5) on chr.1 and the lowest (1) on chr.2, chr.5 and chr.7, respectively. One SNP on chr.1 positioned at 63,055,659 (*p*-value = 4.62E-05) contributed 15.05% to phenotypic variation. Ten SNPs were found associated with quercetin on each of chr.2, chr.4, chr.5, chr.7, and chr.11. One SNP on chr.5 positioned at 29,043,577 (*p*-value = 1.01E-05) contributed 21.64% phenotypic variation. For vanillin, 7 SNPs were found significantly associated that are positioned on chr.1, chr.2, chr.4, chr.5 and chr.7. One SNP on chr.4 positioned at 37,569,986 (*p*-value = 3.56E-05) contributed 23.24% phenotypic variation. For catechin, 7 SNPs were significantly associated and are positioned on chr.1, chr.3, chr.4, chr.5 and chr.6. One SNP on chr.3 positioned at 5,955,359 (*p*-value = 8.79E-06) contributed 23.28% phenotypic variation. For diacaffeoyl-hexoside (DH), 5 SNPs were found significantly associated and are positioned on chr.6 and chr.7. One SNP on chr.6 positioned at 31,943,608 (*p*-value = 1.85E-05) contributed 15.87% phenotypic variation. For swertiamacroside isomer (SI), 5 SNPs were found significantly associated that are positioned on chr3, chr4 and chr5. One SNP on chr.5 positioned at 9,638,542 (*p*-value = 6.62E-05) contributed 15.30% phenotypic variation. For quercetin 3-D-glucoside (QDG), 4 SNPs were found significantly associated that are positioned on chr.1, chr.6 and chr.7. One SNP on chr.7 positioned at 36,838,233 (*p*-value = 8.32E-06) contributed 17.41% phenotypic variation. For duratin, 4 SNPs were found significantly associated that are positioned on chr.1, chr.3 and chr.5. One SNP on chr.1 positioned at 41,194,007 (*p*-value = 3.84E-05) contributed 25.26% phenotypic variation. For galloyl ester of 5,6,7-trihydroxy-2,3 dihydrocyclopents(b)chromene-1,9-dione-3-carboxylic acid hexoside (GEDC), 3 SNPs were found significantly associated that are positioned on chr.1, chr.3 and chr.7. One SNP on chr.1 positioned at 19,115,204 (*p*-value = 1.02E-04) contributed 15.81% phenotypic variation. For orientin, 3 SNPs were found significantly associated that are positioned on chr.5 and chr.8. One SNP on chr.8 positioned at 44,828,457 (*p*-value = 3.37E-05) contributed 20.15% phenotypic variation. For catechin glycoside (CG), 3 SNPs were found significantly associated that are positioned on chr.3 and chr.6. One SNP on chr.3 positioned at 5,955,778 (*p*-value = 1.56E-04) contributed 14.75% phenotypic variation. For caffeic acid hexoside (CAH), 3 SNPs were found significantly associated that are positioned on chr.1, chr.2 and chr.3. One SNP on chr.3 positioned at 27,868,660 (*p*-value = 9.36E-05) contributed 14.70% phenotypic variation. For Rutin, 2 SNPs were found significantly associated that are located on chr3 and chr5. and contributed 12.70% and 15.66% phenotypic variation. For kaempferol, 2 SNPs were found significantly associated that are located on chr.1 and chr.5 and contributed 17.69% and 19.87% phenotypic variation. For (epi)afzelchin-(epi) catechin (EAEC), only 1 SNP was found significantly associated that is located on chr.5 and contributed 26.40% to phenotypic variation. For epiafzelchin epicatechin-o-methylgallate (EEM), only 1 SNP was found significantly associated that is located on chr.1 and contributed 23% to phenotypic variation. For UNK2, only 1 significantly associated SNP was found on chr.2 and contributed 17.11% to phenotypic variation.

### LD Plot and haplotype blocks

LD was calculated from 4,142,684 pairs using 100 markers sliding window operation, out of which 8% was with zero LD and 23% was found in the significant range (*p*-value < 0.05). As the physical distance increases, the r^2^ distribution showed a rapid LD decay for all genotypes. A total of 1783 haplotype blocks were identified, containing 68% markers (Supplementary Figs. 3 and 4). The blocks were uniformly distributed among all chromosomes.

### *In-silico* analysis for candidate gene identification

*In-silico* analysis revealed a total of 168 genic sequences linked with different marker traits (Table 1). The candidate genes were subjected to pathway analysis using the KEGG-KASS server and GO process identification using the Uniprot database to gain insight into the biological process. According to GO analysis, the key biological processes involved are biotic stimulus response, phospholipid biosynthetic process, protein phosphorylation, lipid transport, oxidative stress response, and ion transport (Table 2). The key molecular functions of the identified candidate genes were flavin adenine dinucleotide binding, cysteine-type peptidase activity, protein hetero dimerization and protein binding (Table 2). KEGG-KASS server revealed that the identified candidate genes were related to metabolic pathways of butanoate, glycerol-phospholipids, arachidonic acid, glutathione, alanine, aspartate and glutamate and biosynthetic pathways of secondary metabolites such as flavonoids, sesquiterpenoids and triterpenoids (Table 3).

**Table 2:** Gene ontology annotation of the identified candidate genes

Genes	Gene ID	Name	Group
FtPinG0007389500	GO:0030001	Metal ion transport	Biological process
FtPinG0007897200	GO:0006355	Regulation of transcription, DNA-templated	Biological process
FtPinG0008424800	GO:0008152	Metabolic process	Biological process
FtPinG0006711700	GO:0006508	Proteolysis	Biological process
FtPinG0000116900	GO:0006397	mRNA processing	Biological process
FtPinG0006973100	GO:0055085	Transmembrane transport	Biological process
FtPinG0005258100	GO:0006869	Lipid transport	Biological process
FtPinG0007913300	GO:0009772	Photosynthetic electron transport in photosystem II	Biological process
FtPinG0006589600	GO:0006351	Transcription,DNA-templated	Biological process
FtPinG0009313800	GO:0006952	Defense response	Biological process
FtPinG0006973100	GO:0016020	Membrane	Cellular component
FtPinG0007345300	GO:0016021	Integral component of membrane	Cellular component
FtPinG0009546600	GO:0005741	Mitochondrial outer membrane	Cellular component
FtPinG0000107500	GO:0009579	Thylakoid	Cellular component
FtPinG0009566500	GO:0005737	Cytoplasm	Cellular component
FtPinG0005842300	GO:0000786	Nucleosome	Cellular component
FtPinG0002741100	GO:0003777	Microtubule motor activity	Molecular function
FtPinG0009334000	GO:0003824	Catalytic activity	Molecular function
FtPinG0004200100	GO:0005488	Binding	Molecular function
FtPinG0009099100	GO:0005515	Protein binding	Molecular function
FtPinG0001650100	GO:0016491	Oxidoreductase activity	Molecular function
FtPinG0007484300	GO:0005506	Iron ion binding	Molecular function
FtPinG0004748800	GO:0003676	Nucleic acid binding	Molecular function
FtPinG0007147500	GO:0010277	Chlorophyllide a oxygenase [overall] activity	Molecular function
FtPinG0008486000	GO:0003924	GTPase activity	Molecular function
FtPinG0006758300	GO:0008168	Methyltransferase activity	Molecular function
FtPinG0007606600	GO:0003743	Translation initiation factor activity	Molecular function
FtPinG0001108200	GO:0004602	Glutathione peroxidase activity	Molecular function
FtPinG0006832800	GO:0008444	CDP-diacylglycerol-glycerol-3-phosphate3-phosphatidyltransferase activity	Molecular function
FtPinG0009552900	GO:0016788	Hydrolase activity, acting on ester bonds	Molecular function
FtPinG0009047000	GO:0003723	RNA binding	Molecular function
FtPinG0006470900	GO:0000287	Magnesium ion binding	Molecular function
FtPinG0008592100	GO:0003700	DNA-binding transcription factor activity	Molecular function
FtPinG0003394400	GO:0003779	Actin binding	Molecular function
FtPinG0007781400	GO:0004672	Protein kinase activity	Molecular function
FtPinG0007295400	GO:0003677	DNA binding	Molecular function
FtPinG0009047800	GO:0005524	ATP binding	Molecular function
FtPinG0007298300	GO:0004252	Serine-type endopeptidase activity	Molecular function
FtPinG0007914400	GO:0008137	NADH dehydrogenase (ubiquinone) activity	Molecular function

**Table 3:** The description of identified genes related to different metabolic pathways

S.No	Pathway name and ID	Enriched genes	Pathway function
1	Metabolic pathway ko01100	• Glutathione peroxidise • CDP-diacylglycerol-glycerol-3-phosphte-3-Phosphatidyltransferase • ATP citrate (pro-s)-lyase • 3-methylcrotonyl-coA carboxylase alpha subunit • F-type H + /Na + -transporting ATPase subunit beta Photosystem I P700 chlorophyll a apoprotein A1Photosystem II P680 reaction center D1 protein • NAD(P)H-quinone oxidoreductase subunit 5 (3S,6E)-nerolidolsynthase • Prenylcysteine alpha-carboxyl methylesterase • Succinate-semialdehyde dehydrogenase, mitochondrial Cinnamyl-alcohol dehydrogenase	Converts sugar, into more readily usable materials. These reactions occur inside of a cell, where enzymes, or protein molecules, break down or build up molecules
2	Biosynthesis of secondary metabolites ko01110	• ATP citrate (pro-S)-lyase • Leucoanthocyanidin reductase • Prenylcysteine alpha-carboxyl methylesterase • Cinnamyl-alcohol dehydrogenase	These compounds induce stress onto a plant leading to increased production of secondary metabolites
3	Butanoatemetabolism ko01120	Succinate-semialdehyde dehydrogenase, mitochondrial	Butanoate metabolism describes the metabolic fate of a number of short chain fatty acids or short chain alcohols
4	Oxidative phosphorylation ko00190	• F-type H + /Na + -transporting ATPase subunit beta • NAD(P)H-quinone oxidoreductase subunit 5	Oxidative phosphorylation is the principal purpose of oxygen respiration and for the generation of energy in the body
5	Photosynthesis ko00195	• F-type H + /Na + -transporting ATPase subunit beta Photosystem I P700 chlorophyll a apoprotein A1 • Photosystem II P680 reaction center D1 protein	To create oxygen and energy in the form of sugar
6	Glycerophospholipidmetabolism ko00564	CDP-diacylglycerol–-glycerol-3-phosphate3-phosphatidyltransferase	The metabolites of glycerophospholipid pathway probably maintained the stability of cell membranes against hypoxic stress to relieve the cell injury
7	Arachidonic acid metabolism ko00590	Glutathione peroxidase	Arachidonic acid metabolism provides a pathway for the generation of diverse, fast-acting, short-lived signaling molecules
8	Alanine, aspartate and glutamate metabolism ko00250	Succinate-semialdehyde dehydrogenase, mitochondrial	The defense compound that enables plants to withstand various stresses such as hypoxia, waterlogging and drought, and indirectly as a precursor to the compounds pantothenate and CoA, and position in amino acid metabolism
9	Valine, leucine and isoleucine degradation ko00280	3-methylcrotonyl-CoA carboxylase alpha subunit	They are needed for the physiological response to stress, in energy production, and particularly for the normal metabolism
10	Glutathione metabolism ko00480	Glutathione peroxidase	Glutathione plays important roles in antioxidant defense, nutrient metabolism, and regulation of cellular events
11	Terpenoid backbone biosynthesis ko00900	Prenylcysteine alpha-carboxyl methylesterase	The terpenoid backbone biosynthesis pathway is responsible for the synthesis of different backbones for terpenoids; (E)-β-farnesene (EβF), a sesquiterpene, is the major component of aphid alarm pheromone
12	Sesquiterpenoid and triterpenoid biosynthesis ko00909	(3S,6E)-nerolidol synthase	A group of terpenoids consisting of three isoprene units and are derived from farnesyl diphosphate (FPP) and can be cyclized to produce various skeletal structures
13	Flavonoid biosynthesis ko00941	Leucoanthocyanidin reductase	Major class of plant secondary metabolites that serves a multitude of functions including pigments and antioxidant activity. Flavonoids are synthesized from phenylpropanoid derivatives by condensation with malonyl-CoA

## Discussion

The enormous diversity of structurally distinct metabolites found in the plant metabolome are genetically controlled. It has been hypothesised that species-level metabolome variations are significantly more extensive than previously believed [17]. This calls for the integration of metabolomics and genetics techniques like QTL and GWAS for examining the genetic control of the metabolome and enabling the delineation of metabolic pathways and the dissection of agronomic features [18–20]. Most of the reported metabolites are necessary for the plant's survival, as well as for its ability to grow and interact with its environment [21]. Some of these metabolites confer special nutritional benefits to crops like buckwheat [22]. Our endeavour to characterise buckwheat secondary metabolites was based on the premise that these compounds provide an effective way of defence against biotic and abiotic challenges as well as contribute to the nutritional quality of this valuable crop [23, 24].

Metabolomic profiling has been frequently used in conjunction with genetic techniques like genome-wide association studies (GWAS as mGWAS) and quantitative trait loci (QTL as mQTL) to discover the functional genes behind the variation in metabolite content of different plant species. Tomato and *Arabidopsis* were the first plants wherein mQTL tools were applied previously [25, 26]. Following these ground-breaking researches, the mQTL technique was widely modified to identify the various genetic components controlling the metabolome in many plant species including the Tartary buckwheat [27, 28], offering insights into the genetic and biochemical underpinnings of metabolic pathways. An obvious advantage of using metabolomics is the complexity of phenotyping as well as difficulties associated with large scale field phenotyping. The relative levels of numerous metabolites can be comprehensively profiled with ease and associated to the phenotypes of interest either directly or indirectly [29]. Therefore, identifying putative functional genes governing the variation in metabolite concentration may aid in our understanding of important crop characteristics. A huge population's genes can be identified using GWAS at substantially higher mapping resolutions [14]. In rice, the metabolite trigonelline (N-methyl nicotinic acid) implicated in grain width with its underlying genetic factors were discovered using the same approach.

The current study focuses on the use of mGWAS to identify candidate genes and metabolic pathways in buckwheat. Metabolic pathways are composed of highly varied yet vaguely linked metabolites, which could be thought of as chemical decorations on a number of fundamental structures [30]. Here we not only reported metabolomic profiling data from buckwheat seed but also discovered high-confidence candidate genes. The ultimate objective of this study is the understanding of the genetics underlying traits of interest, which in turn may benefit breeding efforts that seek improvement in economically important traits in buckwheat. The current study used genome-wide association to identify 99 significant markers underlying the studied traits. We were able to predict important genes that encode metabolic and biosynthetic pathways. An enriched gene involved in the flavanoid biosynthesis pathway was also discovered. These findings are significant because flavonoids are the largest class of secondary metabolites found in plants and have a wide range of functional roles, including pigments and antioxidant properties [30]. In addition to flavonoids, different components of amino acid metabolism pathways of alanine, aspartate, glutamate, glutathione, valine, leucine and isoleucine were identified. These defence compounds help plants to withstand various stresses such as hypoxia, water logging, and drought, and act indirectly as a precursors to the compounds pantothenate and CoA, as well as play a central role in amino acid metabolism. They have been implicated in physiological response to stress, normal metabolism and energy processes such as photosynthesis and respiration. Similarly, glutathione is crucial for the regulation of cellular processes, nutrition metabolism, and antioxidant defence. To that end, our data not only provides specific candidate genes as molecular resources that can be effectively used after validation, but also enlightens further metabolite network exploration (Supplementary Figs. 5 and 6). The information generated in the present study will surely facilitate the metabolomics-associated breeding of buckwheat in the future.

## Conclusion

The present study exemplified the potential of integrating GBS technology with metabolomics that led to the discovery of a significant number of potential SNP markers for association mapping and is a valuable resource for QTL studies for the breeding programmes. As such, 68 common QTLs identified in this study by using *F. tataricum* and *F. esculentum* reference genomes might have better implications in improving metabolome content in both common and Tartary buckwheat through molecular breeding approach.

Additionally, the identified candidate genes with potential roles can be explored further through more extensive research. The findings of this study will promote the efficient use of genetic and genomic resources aimed to raising the yield potential and enhancing the metabolite contents and overall quality of buckwheat.

## Methods

### Plant material

A total of 130 diverse buckwheat genotypes were used as plant material in the present study (Supplementary Table 3). Buckwheat germplasm was collected from different geographical regions of Western Himalayan state of Jammu and Kashmir, India and some of the genotypes were also procured from National Bureau of Plant Genetic Resources (NBPGR), New Delhi (Supplementary Fig. 7). Most of the collected genotypes were maintained in the crop research fields of Sher-e-Kashmir University of Agricultural Sciences & Technology of Kashmir, India.

### Quali-quantitative characterization of phenol secondary metabolites

Phytochemical characterization of buckwheat samples was performed by HPLC–DAD-MS. Samples were prepared by extracting 200 mg of dried powdered samples in 25 mL of a 50:50 *v/v* methanol:water mixture, using an ultrasound bath to increase the extraction efficiency (20 min at r.t.). After centrifugation at 13,000 rpm for 10 min, supernatant was collected and directly injected in the instrument for analysis.

Chromatographic separation was performed using a Phenomenex Synergy MAX-RP 80A (4 µm, 150 × 2.0 mm) column as stationary phase, and a mixture of 1% formic acid in water (A) and acetonitrile (B) as mobile phase. Elution gradient was set to the following setting: 0 min, 95% A; 10 min, 50% A; 13 min, 50% A; 18 min, 10% A; 19 min, 10% A; 20 min, 95% A. Column was left to equilibrate for 5 min. Flow rate was 0.4 mL/min with injection volume set to10 µL.

Identification of eluted compounds was performed using integrated DAD and MS data. UV–Vis absorbance was monitored in the range 200–600 nm, and the spectrum of each eluted compound was used to determine its chemical class. Regarding MS, fragmentation data of each compound obtained from MS^n^ experiments were compared with literature data to identify the eluted compounds. The following conditions was set for MS: needle voltage- 4500 V; capillary voltage- 70 V; RF loading- 100%; nebulising gas pressure- 20 psi (nitrogen); drying gas pressure- 15 psi; drying gas temperature- 350 °C. and mass range was 50–2000 Da. Fragmentation patterns of eluted compounds were obtained using the turbo detection data scanning (TDDS®) function of the instrument, setting n = 4 levels of fragmentation.

For the quantification of phenolic compounds from buckwheat samples, standard calibration curves built from DAD measurements were used. Flavonoids were quantified using rutin as standard compound (1-100 µg/mL in methanol), and calibration curve (*y* = 51.52*x* – 183.22; R^2^ = 0.999) was built by monitoring the absorbance values of standard solutions at λ_max_ = 350 nm. For phenolic acids, chlorogenic acid was used as reference compound (0.9–90 µg/mL in methanol), and the calibration curve (*y* = 90.54*x* – 32.84; R^2^ = 0.999) was built monitoring the absorbance values of standard solutions at λ_max_ = 280 nm. Standard solutions (1–100 µg/mL in methanol) of gallic acid were used for the quantification of gallic derivatives, and the calibration curve was built at λ_max_ = 280 nm: *y* = 109.72*x* – 68.33; R^2^ = 0.999. Finally, catechin derivatives were quantified using a catechin calibration curve (*y* = 20.81*x* – 29.61; R^2^ = 0.999) and was built analysing the absorbance value at λ_max_ = 280 nm of catechin solutions (methanol) in the concentration range of 1–100 µg/mL.

### Metabolomics analysis

Metabolomics exploration of buckwheat samples dataset was performed using the Metaboanalyst v. 5.0 platform [31]. For this, quali-quantitative chemical data were organized in a proper data matrix and submitted to the web platform. Data were log transformed and Pareto scaled before analysis. This was accomplished through the use of both unsupervised Principal Component (PCA) and heatmap analyses, as well as supervised methods such as Partial Least Squares Discriminant Analysis (PLS-DA). To avoid over fitting of results, PLS-DA models were validated by using both permutation test (1000 random permutations) and leave-one-out cross validation, whose R^2^, Q^2^ and accuracy parameters were used to assess the robustness and predictability of the models. Variables (metabolites) significantly associated to specific sample groups were selected by using a Volcano plot, setting as threshold values FDR-adjusted p-value < 0.05 and Fold Change > 2.

### DNA extraction, library preparation and sequencing

Seeds of 130 diverse genotypes of buckwheat was sown in plastic trays for three weeks in a polyhouse and the harvested shoots were used for genomic DNA extraction using CTAB method and the quality as well as quantity of DNA was checked on both gel electrophoresis (0.8% Agarose) and nano-drop (mySPEC, Wilmington, USA). GBS libraries were prepared following the method reported in [32], with minor modification. 20 µL digestion reaction contained 1X NEB Buffer, 3.6 U ApeKI and 100 ng of DNA was digested for 4 h at 75 °C. The barcoded adapters were then ligated to sticky ends by using T4 ligase (New England Biolabs). To inactivate the T4 ligase, samples were incubated at 22 °C for 1 h before being heated to 65 °C for 30 min. The sets of 130 digested DNA samples were combined (5 µL each), each with a different barcode adapter, and purified using a commercial kit (QIAquick PCR Purification Kit; Qiagen, Valencia, CA) according to the manufacturer's instructions. DNA samples were eluted in a 25 µL final volume.

Restriction fragments from each library were then amplified in 50 µL volumes containing 10 µL pooled DNA fragments, 25 µl of KAPA HiFi Hot Start Ready Mix PCR, and 1 µL each of the P5 and P7 dual indexing primers (12.5 pmol). These primers included complementary sequences for priming future DNA sequencing reactions, attaching PCR products to oligonucleotides that coat the Illumina sequencing flow cell, and amplifying restriction fragments with ligated adapters. With 0.9X AMPure XP beads (Catalog: A63881, Beckman Coulter), the final PCR products were purified to get rid of any primers that weren't used. The final 130-plex DNA library that had been purified was measured using an Agilent Bioanalyzer before being sequenced on an Illumina HiSeqTM X10 platform (Illumina® Inc., San Diego, CA, USA) using V4 sequencing chemicals.

### Post-sequencing analysis

The raw reads were filtered for adapter sequences, low quality reads and low-quality residues towards 5` region of the sequence. After quality filtering and data de-multiplexing, the high-quality sequences were mapped to the Tartary buckwheat reference genome assembly (GCA 002319775.1; http://www.mbkbase.org/Pinku1/) using BWA program V 0.7.5 [33]. SNPs were mined from the coding and non-coding regions and were subsequently annotated. The SNPs were annotated to the genic, intergeneic, non-coding and regulatory regions using SNPEFF program [34].

Moreover, a comprehensive comparison of the genetic sequences at the genomic level between *F. esculentum* (Common Buckwheat) and *F. tataricum* (Tartary Buckwheat) was performed through pairwise genome alignment, using GSALIGN program (https://github.com/hsinnan75/GSAlign). This process aimed to elucidate the shared characteristics and distinctions within the genomes of these two buckwheat species. The pairwise genome alignment between the two buckwheat genomes encompassed a series of steps, ensuring accurate and reliable results. Initially, the genomic data of both species underwent a pre-processing stage to eliminate any extraneous elements that might introduce noise and potentially hinder the alignment process. By reducing unwanted artefacts, the subsequent alignment was enhanced, allowing for more precise comparison of the genetic sequences. To optimize the alignment, GSALIGN tends to maximise the similarity between corresponding regions while minimizing any gaps that might occur in the alignment. By strategically aligning the sequences, the software facilitated the identification and comparison of specific genetic elements shared between the two species. The results were visualized using DotPlot (https://dotplot.soft112.com/).

### Population structure analysis

Population structure was estimated using a Bayesian Markov Chain Monte Carlo model (MCMC) implemented in STRUCTURE v2.3.4 [35]. The filtered SNPs were converted to structure format using PGD Spider version 2.1.1.5. For each population (k) set number from 2 to 7, three runs were completed. For each run, the burn-in period and the MCMC replication number were set to 100,000 and 300,000, respectively. Structure Harvester used the log probability of the data [LnP(D)] and delta K (K) based on the rate of change in [LnP(D)] between subsequent populations to estimate the most likely K-value [36]. The neighbour-joining tree was built using Phylip and MEGA5 [37].

### Principal component analysis

PCA was calculated using PLINKV 1.9 [38] and then plotted by using R program. Dendrogram analysis was done using TASSEL V4 using Neighbour-Joining method and then plotted with Structure Q-matrix using iTOL. PCA plot was made on four populations which were detected using Structure. Using high quality SNPs, the population structure was graded for K-values ranging from 1 to 12 across the panel.

### Marker trait association

GAPIT V3, an R package that conducts a Genome-Wide Association Study (GWAS) and genome prediction, was used to implement the Compressed Mixed Linear Model (CMLM) [39]. Modern statistical genetics tools including the unified mixed model, EMMA, compressed mixed linear model, and P3D/EMMAx are used in this application. SNPs were considered significant using threshold log10 (*p*-value) < 1E-4. Manhattan plots and quantile–quantile (QQ) plots were developed using R-package QQMAN. Manhattan plots revealed statistically significant associated markers, and quantile–quantile (QQ) plots were created to graphically depict the associated marker distribution pattern. GAPIT was used to calculate the R squared values (r2) for markers; the r2 value explains the proportion of phenotypic variation explained by each SNP locus.

### LD plots and haplotype blocks

Linkage disequilibrium (LD) was measured by the parameter r^2^ using SNPs with high confidence. The values were calculated using TASSEL v5.0 and the values were plotted against genetic distance (in bp) in R software [40]. A threshold of r^2^ = 0.2 was used to determine LD extent. The size of LD blocks was determined by fitting the second LOESS decay curve to the r^2^ values plotted against the physical distance among markers. Using the Gabriel et al. 2002 [41] described confidence interval; Haploview 4.2 was used to identify haplotype blocks from the entire set of SNPs [42]. The analysis excluded heterozygous loci.

### Candidate gene identification

The gene containing the SNP was used to determine the probable candidate gene search from the significant SNP-trait associations obtained from mGWAS using the SNPEFF programme V5.1 against *F. esculentum* annotation downloaded from NCBI [34]. The candidate genes were mapped to the Kyoto Encyclopaedia of Genes and Genomes (KEGG) database using the KEGG-KAAS (KEGG Automatic Annotation Server) server for pathway analysis and Gene Ontology (GO) annotation was carried out using standalone BLASTP and BLASTX [43] against the Uniprot database (release 2022_02) to gain insight into the functional role of candidate genes with SNPs (UniProt Consortium, 2019).

## Supplementary Information

**Additional file 1: Table ST1.** Qualitative results obtained from the HPLC–DAD-MS analysis of Buckwheat samples. **Table ST2.** Summary of number of effects by type and region. **Table ST 3.** List of buckwheat genotypes isolated from India and used in present study.

**Additional file 2: SF 1.** Chromatogram obtained from the HPLC–DAD analysis of Buckwheat samples. **SF 2.** Pair-wise alignment between common (y-axis) and Tatarian buckwheat (x-axis). **SF 3.** LD Plot across the 8 buckwheat chromosomes. **SF 4.** Haplotype blocks of 8 buckwheat chromosomes. **SF 5.** Simple illustration of associated biological process of identified genes. **SF 6.** Simple illustration of molecular functions of identified genes. **SF 7.** Map showing different collection locations of buckwheat germplasm. The images were obtained from goggle map version 2.1 and are available at https://www.google.co.in/maps/@34.1508271,74.8857874,15z?hl=en&authuser=0.

## Data Availability

The datasets used and analysed during the current study will be available from the corresponding author on reasonable request.

## Abbreviations

GBS: Genotyping-by-Sequencing
GAPIT: Genome Association and Prediction Integrated Tool
SNPs: Single Nucleotide Polymorphisms
QTLs: Quantitative Trait Loci
mGWAS: Metabolome Based Genome Wide Association Study
mQTL: Metabolomic Quantitative Trait Loci
HPLC–DAD-MS: High-Performance Liquid Chromatography coupled with Diode-Array Detection and electro-spray ionization tandem mass spectrometry
PCA: Principal Component Analysis
FDR: False Discovery Rate
LD: Linkage disequilibrium
KEGG: Kyoto Encyclopedia of Genes and Genomes
GO: Gene Ontology
PLS-DA: Partial Least Squares Discriminant Analysis
