# Supplementary material for: Metabolic-GWAS provides insights into genetic architecture of seed metabolome in buckwheat
Source: BMC Plant Biol. 2023 Jul 28;23:373. doi: 10.1186/s12870-023-04381-x (PMC10375682; doi:10.1186/s12870-023-04381-x)
Supplement: Supplementary file 1 — Additional file 1: Table ST1. Qualitative results obtained from the HPLC–DAD-MS analysis of Buckwheat samples. Table ST2. Summary of number of effects by type and region. Table ST 3. List of buckwheat genotypes isolated from India and used in present study. [file 12870_2023_4381_MOESM1_ESM.docx]

**Supplementary Tables**

**Table ST 1: Qualitative results obtained from the HPLC-DAD-MS analysis of Buckwheat samples**

| **Chemical class** | **RT (min)** | **m/z** | **MS2** | **MS3** | **Tentative identification** |
| --- | --- | --- | --- | --- | --- |
| **Phenolic acids** | 2.1 | 503 | 341 |  | Dicaffeoylhexoside |
|  | 2.87 | 341 | 179 |  | Caffeic acid hexoside |
|  | 3.45 | 385 | 339 | 193 179 161 | Ferulic acid rhamnosyl derivative |
|  | 3.7 | 315 | 153 | 109 108 | 3, 4-Dihydroxybenzoic acid-O-glucoside |
|  | 6.6 | 487 | 179 |  | Swertiamacroside isomer |
|  | 8.59 | 167 | 152 123 108 |  | Vanillin |
|  |  |  |  |  |  |
| **Flavonoids** | 8.4 | 561 | 289 |  | (epi)afzelchin-(epi)catechin |
|  | 8.6 | 447 |  |  | Orientin |
|  | 8.9 | 609 | 301 |  | Rutin |
|  | 9.2 | 331 | 301 |  | Duartin |
|  | 9.3 | 463 | 301 |  | Quercetin 3-β-D-glucoside |
|  | 10.3 | 727 | 289 |  | Epiafzelchin-epicatechin-O-methyl gallate |
|  | 11 | 741 | 469 272 |  | Epiafzelchin–epicatechin-O-dimethylgallate |
|  | 11.7 | 301 | 151 179 |  | Quercetin |
|  | 11.9 | 319 | 193 |  | Dihydromyricetin |
|  | 12.8 | 285 | 255 229 187 |  | Kaeampferol |
|  |  |  |  |  |  |
| **Catechins** | 6 | 451 | 289 |  | Catechin glycoside |
|  | 7.4 | 289 | 221 245 |  | Catechin |
|  | 7.8 | - |  |  | UNK catechin derivative |
|  |  |  |  |  |  |
| **Gallic acid derivatives** | 4.1 | 197 | 153 |  | Syringic acid |
|  | 4.7 | - |  |  | UNK gallic acid derivative |
|  | 11.03 | 605 | 291 |  | Galloyl ester of 5,6,7-trihydroxy- 2,3-dihydrocyclopenta[b]chromene-1,9-dione-3-carboxylic acid hexoside |

**Table ST 2: Summary of number of effects by type and region**

| **Type** | | | **Region** | | |
| --- | --- | --- | --- | --- | --- |
| **Type** | **Count** | **Percent** | **Type** | **Count** | **Percent** |
| 3ˊ UTR variant | 25767 | 0.377% | Downstream | 1272762 | 18.652% |
| 5ˊ UTR premature start codon gain variant | 4614 | 0.067% | Exon | 259,178 | 3.798% |
| 5ˊ UTR Truncation | 1 | 0% | Gene | 1 | 0% |
| 5ˊ UTR Variant | 35853 | 0.524% | Intergenic | 3605452 | 52.838% |
| Conservative inframe deletion | 268 | 0.004% | Intron | 263.890 | 3.867% |
| Conservative inframe insertion | 310 | 0.005% | Splice site acceptor | 1387 | 0.02% |
| Disruptive inframe deletion | 440 | 0.006% | Splice site donor | 1162 | 0.017% |
| Disruptive inframe insertion | 316 | 0.005% | Splice site region | 12,396 | 0.182% |
| Downstream gene variant | 1272762 | 18.603% | Transcript | 65 | 0.001% |
| Exon loss Variant | 1 | 0% | Upstream | 1341,154 | 19.655% |
| Frameshift Variant | 11121 | 0.163% | UTR 3ˊ | 25,755 | 0.377% |
| Gene fusion | 1 | 0% | UTR5ˊ | 40,444 | 0.593% |
| Initiator code variant | 95 | 0.001% |  |  |  |
| Intergenic region | 3,605452 | 52.697% |  |  |  |
| Intergenic Variant | 6 | 0% |  |  |  |
| Intron variant | 276,446 | 4.041% |  |  |  |
| Missense variant | 142,455 | 2.082% |  |  |  |
| Noncoading transcript variant | 59 | 0.001% |  |  |  |
| Splice acceptor variant | 1414 | 0.021% |  |  |  |
| Splice donor variant | 1311 | 0.019% |  |  |  |
| Splice region variant | 15683 | 0.229% |  |  |  |
| Start lost | 215 | 0.003% |  |  |  |
| Start retained variant | 21 | 0% |  |  |  |
| Stop gained | 8598 | 0.126% |  |  |  |
| Stop lost | 6447 | 0.094 |  |  |  |
| Stop retained Variant | 1653 | 0.024 |  |  |  |
| Synonymus variant | 89,336 | 1.306% |  |  |  |
| Upstream gene variant | 1341154 | 19.602% |  |  |  |

**Table ST 3: List of buckwheat genotypes isolated from India and used in present study**

| **S. No.** | **Genotype** | **Species** | **Collection/Procured** | **Longitude**  **(North)** | **Latitude**  **(East)** | **IC/EC Number** |
| --- | --- | --- | --- | --- | --- | --- |
| 1 | BWZ-1 | *Fagopyrum tartaricum* | Gurez | 34.647^0^ | 74.775^0^ | - |
| 2 | BWZ-2 | *Fagopyrum esculentum* | Gurez | 34.647^0^ | 74.775^0^ | - |
| 3 | BWZ-3 | *Fagopyrum tartaricum* | Gurez | 34.647^0^ | 74.775^0^ | - |
| 4 | BWZ-4 | *Fagopyrum tartaricum* | Budnambal/Kupwara | 34.526^0^ | 74.253^0^ | - |
| 5 | BWZ-5 | *Fagopyrum esculentum* | Kargil | 34.558^0^ | 76.134^0^ | - |
| 6 | BWZ-6 | *Fagopyrum esculentum* | NBPGR/NewDelhi | 28.631^0^ | 77.151^0^ | - |
| 7 | BWZ-7 | *Fagopyrum esculentum* | Kargil | 34.558^0^ | 76.134^0^ | - |
| 8 | BWZ-8 | *Fagopyrum tartaricum* | Ladakh | 33.924^0^ | 77.306^0^ | - |
| 9 | BWZ-9 | *Fagopyrum tartaricum* | NBPGR | 28.631^0^ | 77.151^0^ | - |
| 10 | BWZ-10 | *Fagopyrum esculentum* | Inshan/Kishtward | 34.441^0^ | 75.908^0^ | - |
| 11 | BWZ-11 | *Fagopyrum tartaricum* | Wardwan/Kishtward | 33.867^0^ | 75.536^0^ | IC-0637166 |
| 12 | BWZ-12 | *Fagopyrum tartaricum* | Kargil | 34.558^0^ | 76.134^0^ | - |
| 13 | BWZ-13 | *Fagopyrum tartaricum* | Kargil | 34.558^0^ | 76.134^0^ | - |
| 14 | BWZ-14 | *Fagopyrum tartaricum* | Dasi/Gurez | 34.829^0^ | 77.097^0^ | IC-0637167 |
| 15 | BWZ-15 | *Fagopyrum tartaricum* | Dangan/Gurez | 34.665^0^ | 74.675^0^ | - |
| 16 | BWZ-16 | *Fagopyrum tartaricum* | Dangan/Gurez | 34.665^0^ | 74.675^0^ | IC-0637168 |
| 17 | BWZ-17 | *Fagopyrum tartaricum* | Dangan/Gurez | 34.665^0^ | 74.675^0^ | - |
| 18 | BWZ-18 | *Fagopyrum tartaricum* | Dangan/Gurez | 34.665^0^ | 74.675^0^ | - |
| 19 | BWZ-19 | *Fagopyrum tartaricum* | Dangan/Gurez | 34.665^0^ | 74.675^0^ | - |
| 20 | BWZ-20 | *Fagopyrum tartaricum* | Dangan/Gurez | 34.665^0^ | 74.675^0^ | - |
| 21 | BWZ-21 | *Fagopyrum tartaricum* | Dangan/Gurez | 34.665^0^ | 74.675^0^ | IC-0637170 |
| 22 | BWZ-22 | *Fagopyrum tartaricum* | Dangan/Gurez | 34.665^0^ | 74.675^0^ | IC-0637170 |
| 23 | BWZ-23 | *Fagopyrum tartaricum* | Dangan/Gurez | 34.665^0^ | 74.675^0^ | IC-0637171 |
| 24 | BWZ-24 | *Fagopyrum tartaricum* | Dangan/Gurez | 34.665^0^ | 74.675^0^ | IC-0637172 |
| 25 | BWZ-25 | *Fagopyrum tartaricum* | Karakbal/Gurez | 34.632^0^ | 74.707^0^ | IC-0637173 |
| 26 | BWZ-26 | *Fagopyrum tartaricum* | Karakbal/Gurez | 34.632^0^ | 74.707^0^ | IC-0637174 |
| 27 | BWZ-27 | *Fagopyrum tartaricum* | Dasi/Gurez | 34.665^0^ | 74.612^0^ | IC-0637175 |
| 28 | BWZ-28 | *Fagopyrum esculentum* | Surchey/Kargil | 34.586^0^ | 76.124^0^ | - |
| 29 | BWZ-29 | *Fagopyrum esculentum* | Saliskot/Kargil | 34.407^0^ | 76.014^0^ | - |
| 30 | BWZ-31 | *Fagopyrum esculentum* | Budnumbal/Kupwara | 34.404^0^ | 73.943^0^ | IC-0637156 |
| 31 | BWZ-32 | *Fagopyrum tartaricum* | Sukurbuchan/Leh | 34.397^0^ | 76.779^0^ | IC-0637176 |
| 32 | BWZ-33 | *Fagopyrum tartaricum* | Farona/Kargil | 34.341^0^ | 75.975^0^ |  |
| 33 | BWZ-34 | *Fagopyrum esculentum* | Takmachik/Leh | 34.294^0^ | 76.771^0^ | IC-0637157 |
| 34 | BWZ-35 | *Fagopyrum tartaricum* | Sukurbuchan/Leh | 34.397^0^ | 76.779^0^ | IC-0637177 |
| 35 | BWZ-36 | *Fagopyrum tartaricum* | Domkhar/Leh | 34.397^0^ | 76.779^0^ | - |
| 36 | BWZ-37 | *Fagopyrum tartaricum* | Saliskot/Leh | 34.407^0^ | 76.014^0^ | - |
| 37 | BWZ-38 | *Fagopyrum esculentum* | G.M.pora/Kargil | 34.450^0^ | 76.068^0^ | - |
| 38 | BWZ-39 | *Fagopyrum tartaricum* | Farona/Kargil | 34.341^0^ | 75.975^0^ | - |
| 39 | BWZ-40 | *Fagopyrum esculentum* | Sukurbuchan/Leh | 34.397^0^ | 76.779^0^ | IC-0637158 |
| 40 | BWZ-41 | *Fagopyrum tartaricum* | Sukurbuchan/Leh | 34.397^0^ | 76.779^0^ | - |
| 41 | BWZ-42 | *Fagopyrum tartaricum* | Sukurbuchan/Leh | 34.397^0^ | 76.779^0^ | - |
| 42 | BWZ-43 | *Fagopyrum tartaricum* | Domkhar/Leh | 34.397^0^ | 76.779^0^ | - |
| 43 | BWZ-44 | *Fagopyrum esculentum* | Domkhar/Leh | 34.397^0^ | 76.779^0^ | IC-0637159 |
| 44 | BWZ-45 | *Fagopyrum esculentum* | Goma/Minji | 34.567^0^ | 76.116^0^ | IC-0637160 |
| 45 | BWZ-46 | *Fagopyrum esculentum* | Chachathan/Kargil | 34.829^0^ | 77.097^0^ | IC-063716 |
| 46 | BWZ-47 | *Fagopyrum esculentum* | Farona/Kargil | 34.341^0^ | 75.975^0^ | IC-0637162 |
| 47 | BWZ-48 | *Fagopyrum esculentum* | Bhagna/Kargil | 33.338^0^ | 75.855^0^ | IC-0637163 |
| 48 | BWZ-49 | *Fagopyrum esculentum* | Dimji/Kishtward | 33.287^0^ | 75.900^0^ | IC-0637164 |
| 49 | BWZ-50 | *Fagopyrum esculentum* | Sayia/Kishtward | 33.341^0^ | 75.891^0^ | IC-0637165 |
| 50 | BWZ-51 | *Fagopyrum tartaricum* | NBPGR/New Delhi | 28.631^0^ | 77.151^0^ | IC-13140 |
| 51 | BWZ-52 | *Fagopyrum tartaricum* | NBPGR/New Delhi | 28.631^0^ | 77.151^0^ | IC-13143 |
| 52 | BWZ-53 | *Fagopyrum tartaricum* | NBPGR/New Delhi | 28.631^0^ | 77.151^0^ | IC-13413 |
| 53 | BWZ-54 | *Fagopyrum tartaricum* | NBPGR/New Delhi | 28.631^0^ | 77.151^0^ | IC-14494 |
| 54 | BWZ-55 | *Fagopyrum esculentum* | NBPGR/New Delhi | 28.631^0^ | 77.151^0^ | IC-16555 |
| 55 | BWZ-56 | *Fagopyrum tartaricum* | NBPGR/New Delhi | 28.631^0^ | 77.151^0^ | IC-17370 |
| 56 | BWZ-57 | *Fagopyrum esculentum* | NBPGR/New Delhi | 28.631^0^ | 77.151^0^ | IC-17371 |
| 57 | BWZ-58 | *Fagopyrum esculentum* | NBPGR/New Delhi | 28.631^0^ | 77.151^0^ | IC-17372 |
| 58 | BWZ-59 | *Fagopyrum esculentum* | NBPGR/New Delhi | 28.631^0^ | 77.151^0^ | IC-17971 |
| 59 | BWZ-60 | *Fagopyrum esculentum* | NBPGR/New Delhi | 28.631^0^ | 77.151^0^ | IC-18040 |
| 60 | BWZ-61 | *Fagopyrum tartaricum* | NBPGR/New Delhi | 28.631^0^ | 77.151^0^ | IC-18751 |
| 61 | BWZ-62 | *Fagopyrum tartaricum* | NBPGR/New Delhi | 28.631^0^ | 77.151^0^ | IC-18757 |
| 62 | BWZ-63 | *Fagopyrum esculentum* | NBPGR/New Delhi | 28.631^0^ | 77.151^0^ | IC-18801 |
| 63 | BWZ-64 | *Fagopyrum esculentum* | NBPGR/New Delhi | 28.631^0^ | 77.151^0^ | IC-18881 |
| 64 | BWZ-65 | *Fagopyrum tartaricum* | NBPGR/New Delhi | 28.631^0^ | 77.151^0^ | IC-18889 |
| 65 | BWZ-66 | *Fagopyrum tartaricum* | NBPGR/New Delhi | 28.631^0^ | 77.151^0^ | IC-22426 |
| 66 | BWZ-67 | *Fagopyrum tartaricum* | NBPGR/New Delhi | 28.631^0^ | 77.151^0^ | IC-24296 |
| 67 | BWZ-68 | *Fagopyrum tartaricum* | NBPGR/New Delhi | 28.631^0^ | 77.151^0^ | IC-24298 |
| 68 | BWZ-69 | *Fagopyrum tartaricum* | NBPGR/New Delhi | 28.631^0^ | 77.151^0^ | IC-24299 |
| 69 | BWZ-70 | *Fagopyrum tartaricum* | NBPGR/New Delhi | 28.631^0^ | 77.151^0^ | IC-24302 |
| 70 | BWZ-71 | *Fagopyrum esculentum* | NBPGR/New Delhi | 28.631^0^ | 77.151^0^ | IC-25744 |
| 71 | BWZ-72 | *Fagopyrum tartaricum* | NBPGR/New Delhi | 28.631^0^ | 77.151^0^ | IC-25999 |
| 72 | BWZ-73 | *Fagopyrum esculentum* | NBPGR/New Delhi | 28.631^0^ | 77.151^0^ | IC-26549 |
| 73 | BWZ-74 | *Fagopyrum esculentum* | NBPGR/New Delhi | 28.631^0^ | 77.151^0^ | IC-26586 |
| 74 | BWZ-75 | *Fagopyrum tartaricum* | NBPGR/New Delhi | 28.631^0^ | 77.151^0^ | IC-26591 |
| 75 | BWZ-76 | *Fagopyrum tartaricum* | NBPGR/New Delhi | 28.631^0^ | 77.151^0^ | IC-37277 |
| 76 | BWZ-77 | *Fagopyrum tartaricum* | NBPGR/New Delhi | 28.631^0^ | 77.151^0^ | IC-37278 |
| 77 | BWZ-78 | *Fagopyrum esculentum* | NBPGR/New Delhi | 28.631^0^ | 77.151^0^ | IC-37281 |
| 78 | BWZ-80 | *Fagopyrum esculentum* | NBPGR/New Delhi | 28.631^0^ | 77.151^0^ | IC-37284 |
| 79 | BWZ-81 | *Fagopyrum tartaricum* | NBPGR/New Delhi | 28.631^0^ | 77.151^0^ | EC-12537 |
| 80 | BWZ-82 | *Fagopyrum esculentum* | NBPGR/New Delhi | 28.631^0^ | 77.151^0^ | EC-18132 |
| 81 | BWZ-83 | *Fagopyrum tartaricum* | NBPGR/New Delhi | 28.631^0^ | 77.151^0^ | EC-18182 |
| 82 | BWZ-84 | *Fagopyrum esculentum* | NBPGR/New Delhi | 28.631^0^ | 77.151^0^ | EC-18237 |
| 83 | BWZ-85 | *Fagopyrum tartaricum* | NBPGR/New Delhi | 28.631^0^ | 77.151^0^ | EC-18629 |
| 84 | BWZ-86 | *Fagopyrum tartaricum* | NBPGR/New Delhi | 28.631^0^ | 77.151^0^ | EC-18740 |
| 85 | BWZ-87 | *Fagopyrum esculentum* | NBPGR/New Delhi | 28.631^0^ | 77.151^0^ | EC-18781 |
| 86 | BWZ-89 | *Fagopyrum esculentum* | NBPGR/New Delhi | 28.631^0^ | 77.151^0^ | E-18827 |
| 87 | BWZ-90 | *Fagopyrum tartaricum* | NBPGR/New Delhi | 28.631^0^ | 77.151^0^ | EC-99945 |
| 88 | BWZ-91 | *Fagopyrum tartaricum* | NBPGR/New Delhi | 28.631^0^ | 77.151^0^ | EC-99946 |
| 89 | BWZ-92 | *Fagopyrum tartaricum* | NBPGR/New Delhi | 28.631^0^ | 77.151^0^ | EC-99948 |
| 90 | BWZ-93 | *Fagopyrum tartaricum* | NBPGR/New Delhi | 28.631^0^ | 77.151^0^ | EC-104036 |
| 91 | BWZ-94 | *Fagopyrum tartaricum* | NBPGR/New Delhi | 28.631^0^ | 77.151^0^ | EC-104037 |
| 92 | BWZ-95 | *Fagopyrum esculentum* | NBPGR/New Delhi | 28.631^0^ | 77.151^0^ | EC-125935 |
| 93 | BWZ-97 | *Fagopyrum esculentum* | NBPGR/New Delhi | 28.631^0^ | 77.151^0^ | EC-125938 |
| 94 | BWZ-98 | *Fagopyrum esculentum* | NBPGR/New Delhi | 28.631^0^ | 77.151^0^ | E-125939 |
| 95 | BWZ-99 | *Fagopyrum tartaricum* | NBPGR/New Delhi | 28.631^0^ | 77.151^0^ | EC-131622 |
| 96 | BWZ-100 | *Fagopyrum tartaricum* | NBPGR/New Delhi | 28.631^0^ | 77.151^0^ | EC-161415-16 |
| 97 | BWZ-101 | *Fagopyrum esculentum* | NBPGR/New Delhi | 28.631^0^ | 77.151^0^ | EC-213685 |
| 98 | BWZ-102 | *Fagopyrum tartaricum* | NBPGR/New Delhi | 28.631^0^ | 77.151^0^ | EC-21662 |
| 99 | BWZ-104 | *Fagopyrum esculentum* | NBPGR/New Delhi | 28.631^0^ | 77.151^0^ | EC-216631 |
| 100 | BWZ-105 | *Fagopyrum esculentum* | NBPGR/New Delhi | 28.631^0^ | 77.151^0^ | EC-216634 |
| 101 | BWZ-107 | *Fagopyrum esculentum* | NBPGR/New Delhi | 28.631^0^ | 77.151^0^ | EC-218734 |
| 102 | BWZ-108 | *Fagopyrum esculentum* | NBPGR/New Delhi | 28.631^0^ | 77.151^0^ | EC-218740 |
| 103 | BWZ-109 | *Fagopyrum esculentum* | NBPGR/New Delhi | 28.631^0^ | 77.151^0^ | EC-218742 |
| 104 | BWZ-110 | *Fagopyrum esculentum* | NBPGR/New Delhi | 28.631^0^ | 77.151^0^ | EC-218784 |
| 105 | BWM-2 | *Fagopyrum tartaricum* | NBPGR/New Delhi | 28.631^0^ | 77.151^0^ | IC-24298 |
| 106 | BWM-9 | *Fagopyrum tartaricum* | NBPGR/New Delhi | 28.631^0^ | 77.151^0^ | IC-26591 |
| 107 | BWM-11 | *Fagopyrum tartaricum* | NBPGR/New Delhi | 28.631^0^ | 77.151^0^ | IC-107960 |
| 108 | BWM-12 | *Fagopyrum tartaricum* | NBPGR/New Delhi | 28.631^0^ | 77.151^0^ | IC-107962 |
| 109 | BWM-13 | *Fagopyrum tartaricum* | NBPGR/New Delhi | 28.631^0^ | 77.151^0^ | IC-107964 |
| 110 | BWM-14 | *Fagopyrum tartaricum* | NBPGR/New Delhi | 28.631^0^ | 77.151^0^ | IC-107967 |
| 111 | BWM-19 | *Fagopyrum tartaricum* | NBPGR/New Delhi | 28.631^0^ | 77.151^0^ | IC-107976 |
| 112 | BWM-21 | *Fagopyrum esculentum* | NBPGR/New Delhi | 28.631^0^ | 77.151^0^ | IC-26549 |
| 113 | BWM-26 | *Fagopyrum esculentum* | NBPGR/New Delhi | 28.631^0^ | 77.151^0^ | IC-107972 |
| 114 | BWM-27 | *Fagopyrum esculentum* | NBPGR/New Delhi | 28.631^0^ | 77.151^0^ | IC-107982 |
| 115 | BWM-29 | *Fagopyrum esculentum* | NBPGR/New Delhi | 28.631^0^ | 77.151^0^ | IC-108501 |
| 116 | BWM-30 | *Fagopyrum esculentum* | NBPGR/New Delhi | 28.631^0^ | 77.151^0^ | IC-108505 |
| 117 | BWM-32 | *Fagopyrum esculentum* | NBPGR/New Delhi | 28.631^0^ | 77.151^0^ | IC-108509 |
| 118 | BWM-33 | *Fagopyrum esculentum* | NBPGR/New Delhi | 28.631^0^ | 77.151^0^ | IC-108511 |
| 119 | BWM-35 | *Fagopyrum esculentum* | NBPGR/New Delhi | 28.631^0^ | 77.151^0^ | EC-18132 |
| 120 | BWM-36 | *Fagopyrum esculentum* | NBPGR/New Delhi | 28.631^0^ | 77.151^0^ | EC-18225 |
| 121 | BWM-37 | *Fagopyrum esculentum* | NBPGR/New Delhi | 28.631^0^ | 77.151^0^ | EC-159498 |
| 122 | BWM-38 | *Fagopyrum esculentum* | NBPGR/New Delhi | 28.631^0^ | 77.151^0^ | EC-218940 |
| 123 | BWM-39 | *Fagopyrum esculentum* | NBPGR/New Delhi | 28.631^0^ | 77.151^0^ | EC-272442 |
| 124 | BWM-40 | *Fagopyrum esculentum* | NBPGR/New Delhi | 28.631^0^ | 77.151^0^ | EC-286379 |
| 125 | BWM-42 | *Fagopyrum esculentum* | NBPGR/New Delhi | 28.631^0^ | 77.151^0^ | EC-323723 |
| 126 | BWM-45 | *Fagopyrum esculentum* | NBPGR/New Delhi | 28.631^0^ | 77.151^0^ | EC-323731 |
| 127 | BWM-46 | *Fagopyrum esculentum* | NBPGR/New Delhi | 28.631^0^ | 77.151^0^ | EC-386667 |
| 128 | BWM-47 | *Fagopyrum esculentum* | NBPGR/New Delhi | 28.631^0^ | 77.151^0^ | EC-386668 |
| 129 | BWM-48 | *Fagopyrum esculentum* | NBPGR/New Delhi | 28.631^0^ | 77.151^0^ | EC-386669 |
| 130 | BWM-49 | *Fagopyrum esculentum* | NBPGR/New Delhi | 28.631^0^ | 77.151^0^ | EC-386671 |
