# Supplementary material for: Metabolic-GWAS provides insights into genetic architecture of seed metabolome in buckwheat
Source: BMC Plant Biol. 2023 Jul 28;23:373. doi: 10.1186/s12870-023-04381-x (PMC10375682; doi:10.1186/s12870-023-04381-x)
Supplement: Supplementary file 2 — Additional file 2: SF 1. Chromatogram obtained from the HPLC–DAD analysis of Buckwheat samples. SF 2. Pair-wise alignment between common (y-axis) and Tatarian buckwheat (x-axis). SF 3. LD Plot across the 8 buckwheat chromosomes. SF 4. Haplotype blocks of 8 buckwheat chromosomes. SF 5. Simple illustration of associated biological process of identified genes. SF 6. Simple illustration of molecular functions of identified genes. SF 7. Map showing different collection locations of buckwheat germplasm. The images were obtained from goggle map version 2.1 and are available at https://www.google.co.in/maps/@34.1508271,74.8857874,15z?hl=en&authuser=0. [file 12870_2023_4381_MOESM2_ESM.docx]

**Supplementary Figures**

**
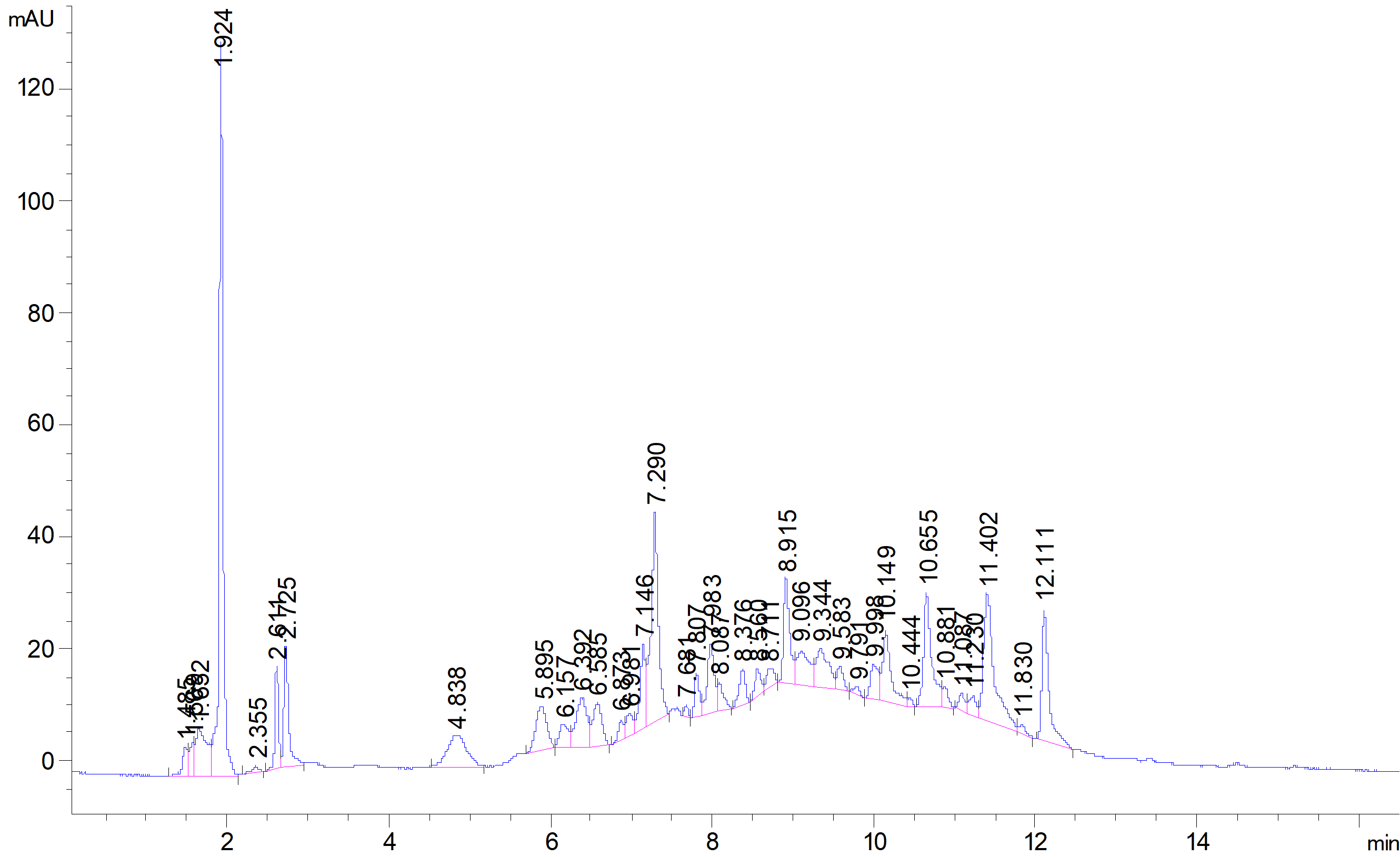
**

**SF 1: Chromatogram obtained from the HPLC-DAD analysis of Buckwheat samples**

**
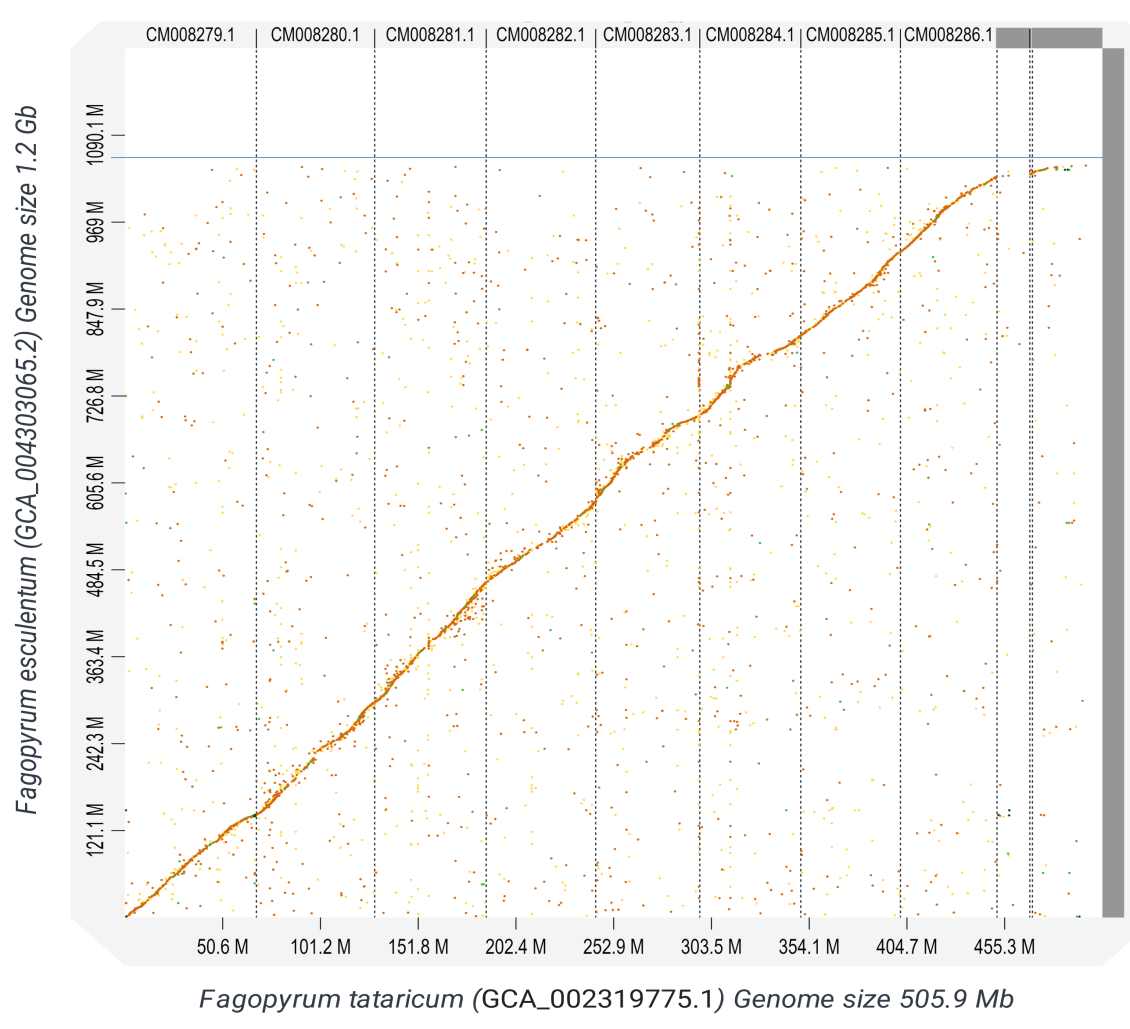
**

**SF 2: Pair-wise alignment between common (y-axis) and Tatarian buckwheat (x-axis).**

**
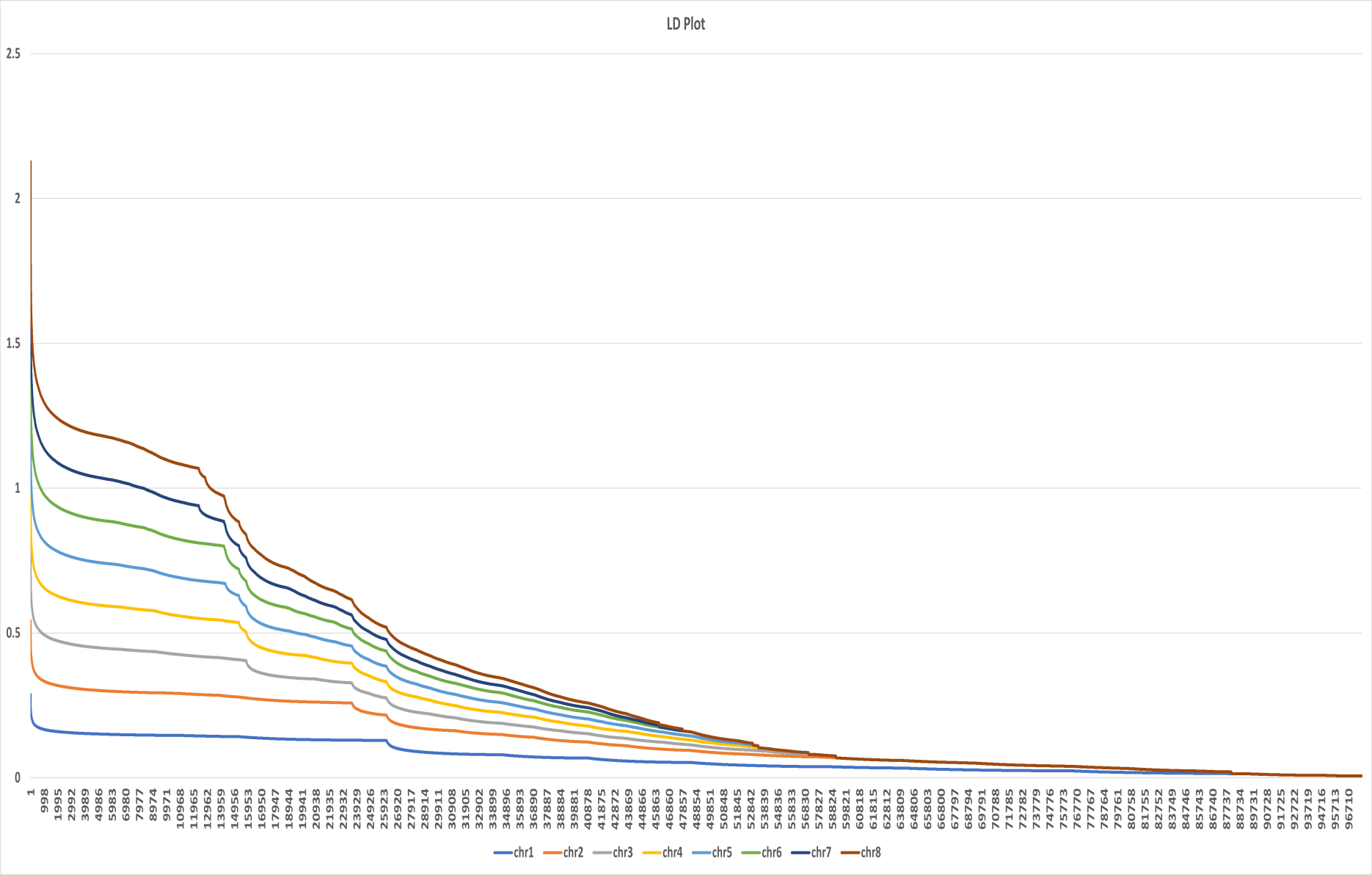
**

**SF 3: LD Plot across the 8 buckwheat chromosomes**

**
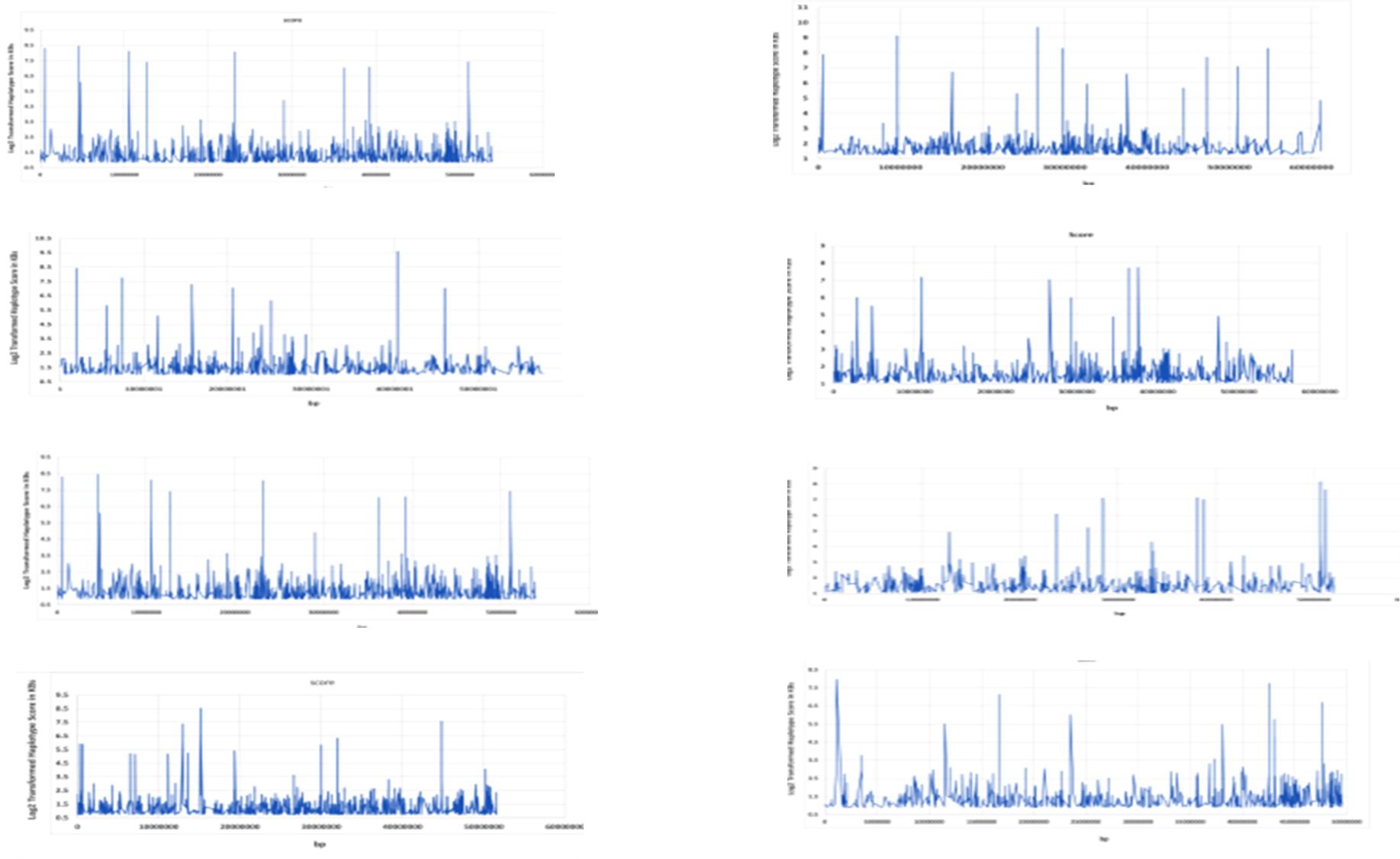
**

**SF 4: Haplotype blocks of 8 buckwheat chromosomes**

**
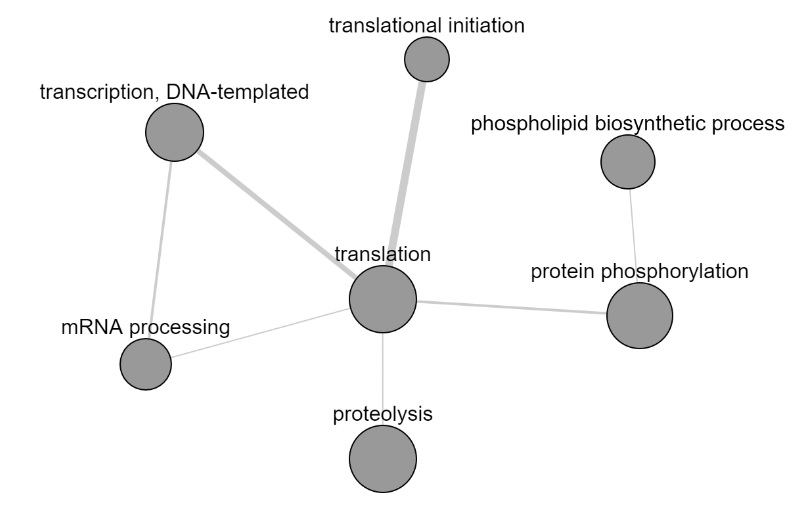
**

**SF 5: Simple illustration of associated biological process of identified genes**

**
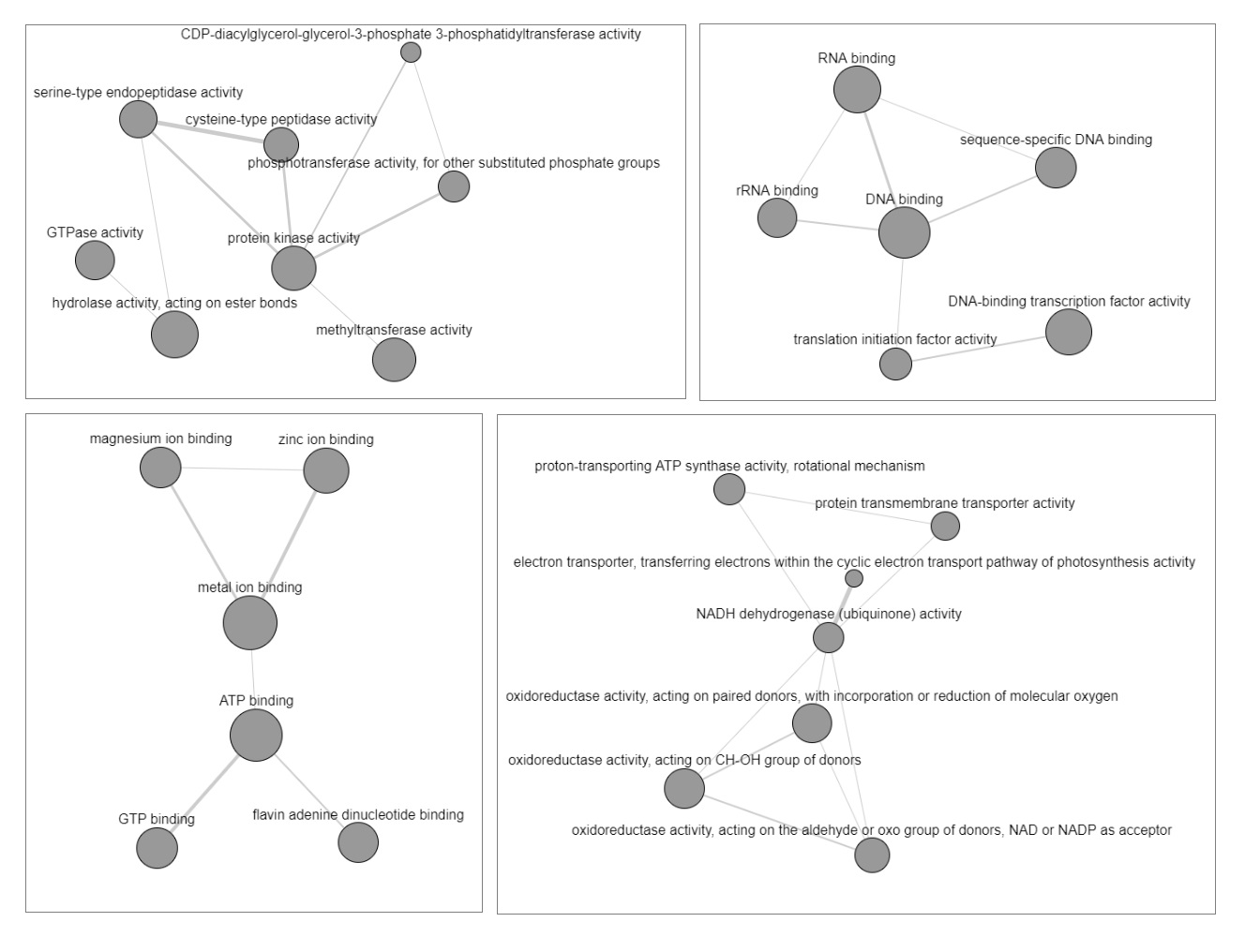
**

**SF 6: Simple illustration of molecular functions of identified genes**

**
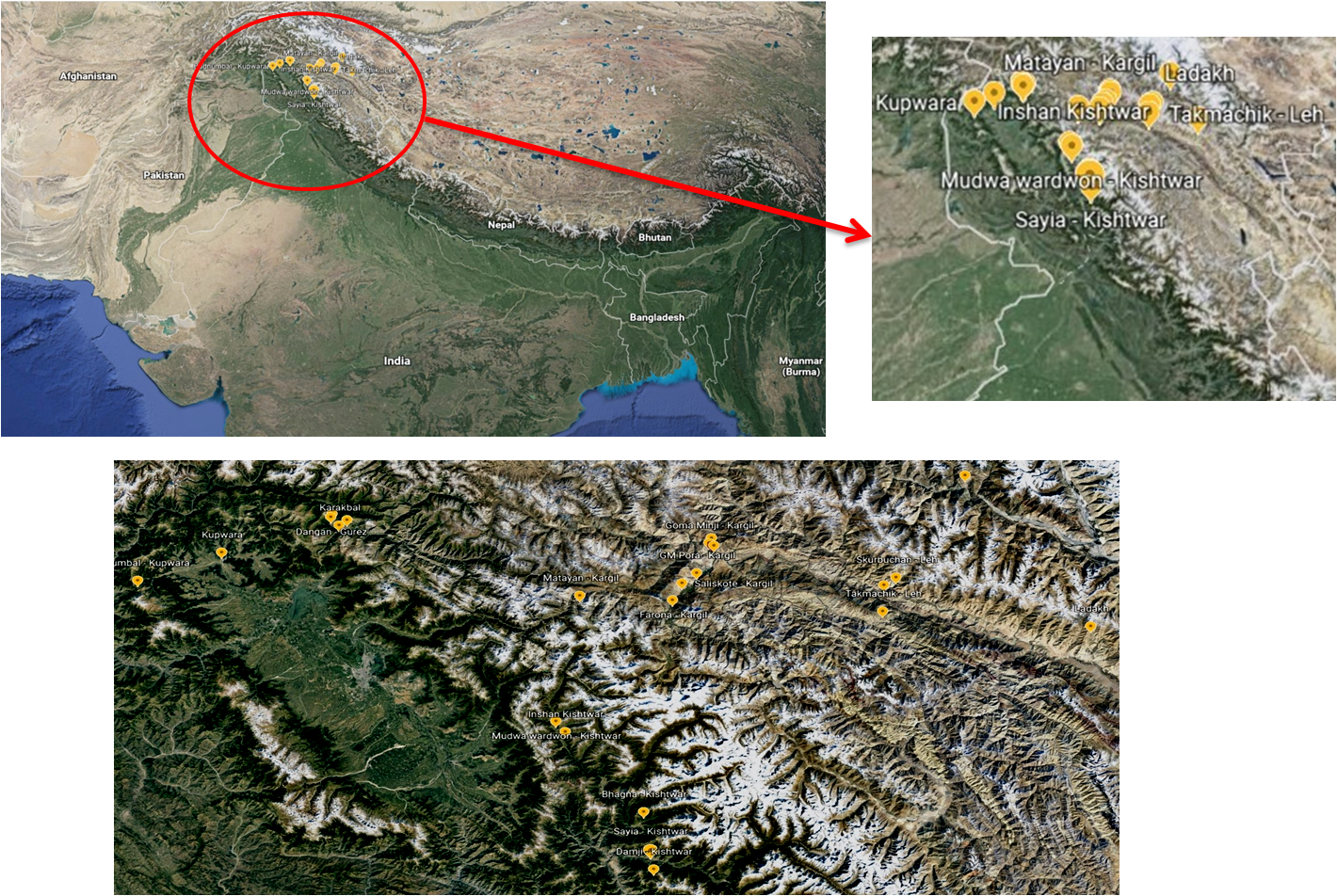
**

**SF 7: Map showing different collection locations of buckwheat germplasm**
